# TMPRSS4 promotes cancer stem–like properties in prostate cancer cells through upregulation of SOX2 by SLUG and TWIST1

**DOI:** 10.1186/s13046-021-02147-7

**Published:** 2021-11-22

**Authors:** Yunhee Lee, Junghwa Yoon, Dongjoon Ko, Minyeong Yu, Soojin Lee, Semi Kim

**Affiliations:** 1 Immunotherapy Research Center, Korea Research Institute of Bioscience and Biotechnology, 125 Gwahak-ro, Yuseong-gu, Daejon, 34141 South Korea; 2 Department of Microbiology and Molecular Biology, Chungnam National University, Daejon, 34134 South Korea; 3 Department of Functional Genomics, Korea University of Science and Technology, Daejon, 34113 South Korea

**Keywords:** TMPRSS4, Stemness, SOX2, SLUG, TWIST1

## Abstract

**Background:**

Transmembrane serine protease 4 (TMPRSS4) is a cell surface–anchored serine protease. Elevated expression of TMPRSS4 correlates with poor prognosis in colorectal cancer, gastric cancer, prostate cancer, non–small cell lung cancer, and other cancers. Previously, we demonstrated that TMPRSS4 promotes invasion and proliferation of prostate cancer cells. Here, we investigated whether TMPRSS4 confers cancer stem–like properties to prostate cancer cells and characterized the underlying mechanisms.

**Methods:**

Acquisition of cancer stem–like properties by TMPRSS4 was examined by monitoring anchorage-independent growth, tumorsphere formation, aldehyde dehydrogenase (ALDH) activation, and resistance to anoikis and drugs in vitro and in an early metastasis model in vivo. The underlying molecular mechanisms were evaluated, focusing on stemness-related factors regulated by epithelial–mesenchymal transition (EMT)-inducing transcription factors. Clinical expression and significance of TMPRSS4 and stemness-associated factors were explored by analyzing datasets from The Cancer Genome Atlas (TCGA).

**Results:**

TMPRSS4 promoted anchorage-independent growth, ALDH activation, tumorsphere formation, and therapeutic resistance of prostate cancer cells. In addition, TMPRSS4 promoted resistance to anoikis, thereby increasing survival of circulating tumor cells and promoting early metastasis. These features were accompanied by upregulation of stemness-related factors such as SOX2, BMI1, and CD133. SLUG and TWIST1, master EMT-inducing transcription factors, made essential contributions to TMPRSS4-mediated cancer stem cell (CSC) features through upregulation of SOX2. SLUG stabilized SOX2 via preventing proteasomal degradation through its interaction with SOX2, while TWIST1 upregulated transcription of *SOX2* by interacting with the proximal E-box element in the *SOX2* promoter. Clinical data showed that *TMPRSS4* expression correlated with the levels of *SOX2*, *PROM1*, *SNAI2*, and *TWIST1*. Expression of *SOX2* was positively correlated with that of *TWIST1*, but not with other EMT-inducing transcription factors, in various cancer cell lines.

**Conclusions:**

Together, these findings suggest that TMPRSS4 promotes CSC features in prostate cancer through upregulation of the SLUG- and TWIST1-induced stem cell factor SOX2 beyond EMT. Thus, TMPRSS4/SLUG–TWIST1/SOX2 axis may represent a novel mechanism involved in the control of tumor progression.

**Supplementary Information:**

The online version contains supplementary material available at 10.1186/s13046-021-02147-7.

## Background

The metastatic cascade is a complex process that consists of multiple important steps: local invasion, intravasation, circulation, extravasation, micrometastasis, and metastatic colonization [1, 2]. As an initial step in cancer metastasis, epithelial tumor cells are induced to invade and disseminate via activation of the epithelial–mesenchymal transition (EMT), a developmental process that is normally observed during embryogenesis and epithelial tissue healing in adulthood [3]. During the EMT, well-polarized epithelial cells gradually lose their epithelial features, such as cell–cell adhesion, and concomitantly acquire certain mesenchymal features such as greater motility and proteolytic activity. The EMT is accompanied by changes at the molecular level: epithelial markers such as E-cadherin are downregulated, and mesenchymal markers such as vimentin are upregulated. These changes are usually mediated, directly or indirectly, by EMT-inducing transcription factors, such as members of the Snail, ZEB, and basic helix-loop-helix (bHLH) families [4–7]. These transcription factors not only induce EMT and cancer cell migration/invasion but also generate cells with self-renewal capacity; elevated resistance to apoptosis, anoikis, and therapeutics; the ability to override senescence; and proangiogenic and proinflammatory activities [4, 6, 8, 9]. However, the molecular mechanisms underlying these changes remain unclear.

SLUG (SNAI2), a member of the Snail family, is upregulated in metastatic breast cancer, colon cancer, lung cancer, mesothelioma, and melanoma [5]. Recently, based on its elevated expression relative to other Snail family members, SLUG was implicated in tumor development and progression of prostate cancer [10]. SLUG is involved in metastatic prostate cancer cell migration and invasion [11], although its role in the regulation of proliferation remains unclear. The bHLH transcription factor TWIST1 is expressed in breast, liver, prostate, gastric, colorectal, and other types of cancers, and its expression is usually associated with invasive and metastatic phenotypes [4, 7]. To induce EMT and invasiveness, TWIST1 directly or indirectly represses E-cadherin transcription through E-box elements in the E-cadherin promoter [7]. SOX2 is a transcription factor that is essential for development and maintenance of stemness. Dysregulation of SOX2 expression contributes to progression of cancers, including prostate cancer. SOX2 promotes cancer cell traits such as proliferation, EMT, migration, invasion, and metastasis, as well as resistance to apoptosis and therapies [12, 13].

Dysregulation of proteases is a hallmark of tumor progression: changes in cell–cell and cell–extracellular matrix (ECM) adhesions are important for cancer metastasis, and proteases play key roles in the modulation of tumor cells and ECM properties. Beyond matrix protein degradation, proteases are involved in all stages of cancer development and progression, including growth, survival, migration, invasion, angiogenesis, and metastasis, through both direct proteolytic activity and regulation of cellular signaling and functions [14, 15]. Over the past two decades, Type II transmembrane proteases (TTSPs) were recognized as a new subfamily of serine proteases. All 17 members of the TTSP subfamily in human share a short cytoplasmic N-terminal domain, a transmembrane domain, a stem region, and a C-terminal proteolytic domain [16–18]. Most TTSPs are overexpressed in a variety of tumors relative to the corresponding normal tissues, indicating their potential as novel markers for tumor development and progression [17]. To date, many studies have analyzed the expression of individual TTSPs during tumor progression and focused on the potential roles of these proteases in tumor cell migration, invasion, and proliferation [17, 19].

TMPRSS4, originally referred to as TMPRSS3 [20], is highly upregulated in a variety of cancers. Elevated TMPRSS4 expression is associated with poor prognosis in non–small cell lung cancer with squamous cell histology, triple-negative breast cancer, cervical cancer, gastric cancer, colon cancer, and prostate cancer [21, 22]. Previously, we reported that TMPRSS4 plays important roles in migration, invasion, and metastasis of human epithelial cancer cells, and that elevated expression of TMPRSS4 is associated with stage progression of colorectal cancer [23–25]. Furthermore, we demonstrated that TMPRSS4 promotes both the invasion and proliferation of cancer cells through activation of AP-1 and subsequent upregulation of SLUG in a manner dependent on MAPKs [22, 26]. These reports suggest that TMPRSS4 may serve as a potential molecular target for anti-cancer therapy.

These previous findings led us to hypothesize that TMPRSS4 drives diverse cellular functions beyond tumor invasiveness during cancer progression. In particular, we postulated that TMPRSS4 contributes to the malignancy of prostate cancer via its tumor-initiation capacity. In this study, we found that TMPRSS4 promoted cancer stem–like properties such as tumorsphere formation, ALDH activation, drug resistance, and anoikis resistance, and subsequent early metastasis (circulating tumor cell (CTC) survival) in prostate cancer cells and upregulated stemness-related markers including SOX2. SLUG and TWIST1 were involved in TMPRSS4-mediated promotion of CSC properties: SLUG stabilized SOX2 by preventing its proteasomal degradation, whereas TWIST1 directly upregulated transcription of *SOX2* through the proximal E-box element in the *SOX2* promoter. These data provide evidence that the TMPRSS4/SLUG–TWIST1/SOX2 axis could be exploited as a target for potential anti-cancer therapy.

## Materials and methods

### Cell lines

PC3, DU145, 22Rv1, LNCaP clone FGC (prostate cancer), HCT-116, and HT-29 (colon cancer) cell lines (American Type Culture Collection (ATCC), Manassas, VA) were maintained in RPMI1640 containing 10% fetal bovine serum at 37 °C in 5% CO_2_. Human embryonic kidney 293E (HEK293E) cells (ATCC) were maintained in Dulbecco’s modified Eagle’s medium (DMEM) with 10% fetal bovine serum. Stable cells (vector-transfected and TMPRSS4-overexpressing cells) established from the PC3 and DU145 cell lines were described previously [22, 27]. Cell were checked for mycoplasma and their identities were confirmed using short tandem repeat (STR)-PCR analysis.

### Transfection with siRNAs, shRNA vectors, and expression vectors

Cells were transfected with siRNA specific to *TMPRSS4* (5′-UCCAGUACGACAAACAGCACGUCUG-3′), *SNAI2* (5′-GGACCACAGUGGCUCAGAA-3′), *TWIST1* (5′-AGCUGAGCAAGAUUCAGACCCUCAA-3′), *BMI1* (5′-GGAGAAGGAAUGGUCCACUUCCAUU-3′), or *SOX2* (5′-CAGUACAACUCCAUGACCA-3′ and 5′-GCUCUUGGCUCCAUGGGUU-3′). siRNA specific to *JUN* or *SP1* was previously described [26]. *TMPRSS4*-specific siRNAs (#665; 5′-TCCAGTACGACAAACAGCACGTCTG-3′ and #368; 5′-GGTTCTCTGCCTGTTTCGACAACTT-3′) and scrambled siRNA (5′-AGACAACGTGTCTTAGAACGCCACC-3′) were subcloned into the pLKO.1 lentiviral shRNA vector (Addgene, Cambridge, MA) digested with AgeI/EcoRI. Cells were transfected with a TMPRSS4-expressing vector pCMV-myc-TMPRSS4 [23], myc-tagged SLUG-expressing vector [22], flag-tagged TWIST1-expressing vector [28], c-Jun-expressing vector [26], or SP1-expressing vector [26]. Transfections were performed using Lipofectamine 2000 (Thermo Fisher Scientific, Waltham, MA). At 48 h after transfection, cells were lysed for analysis of protein and mRNA or harvested for subsequent experiments. Where indicated, cells were co-transfected for 48 h with siRNA and a plasmid. Cells were transfected with siRNA for 42 h and then treated with MG132 (2 μM, Sigma, St Louis, MO) or 0.1% dimethyl sulfoxide (DMSO) for 6 h.

### Immunoblot analysis

Whole-cell lysates were prepared using RIPA buffer as described previously [26] and analyzed using the following primary antibodies: anti-integrin α4, anti-SP1, and anti-GAPDH (Santa Cruz Biotechnology, Dallas, TX); anti-integrin α2 (Chemicon International, Temecula, CA); anti-myc (Upstate Biotechnology, Lake Placid, NY); anti-cyclin D2, anti-phospho-c-Jun(S63), anti-c-Jun, anti-phospho-AKT, anti-AKT, anti-survivin, anti-Bcl-2, anti-SLUG, anti-SOX2, anti-CD133, anti-ABCB1, and anti-KLF4 (Cell Signaling Technology, Danvers, MA); anti-BMI1 (Millipore, Temecula, CA), anti-integrin α5 and anti-E-cadherin (BD Biosciences, San Jose, CA); anti-vimentin and anti-flag (Sigma); anti-TWIST1 (Abcam, Cambridge, MA); and anti-TMPRSS4 (in-house) [26].

### Reverse transcription-quantitative PCR (RT-qPCR)

Total RNA was isolated using TRIzol (Invitrogen, Carlsbad, CA), and cDNA was synthesized using reverse transcriptase (Bioneer, Daejon, Korea). Real-time quantitative PCR was performed using SYBR Green (PKT, Seoul, Korea) on a Rotor-Gene 6000 real-time rotary analyzer (Corbett, San Francisco, CA) with primers specific for *SNAI2* (5′-ATACCACAACCAGAGATCCTCA-3′ and 5′-GACTCACTCGCCCCAAAGATG-3′), *TWIST1* (5′-AAGAGGTCGTGCCAATCAG-3′ and 5′-GGCCAGTTTGATCCCAGTAT-3′), *SOX2* (5′-AATGCCTTCATGGTGTGGT-3′ and 5′-CTTCTCCGTCTCCGACAAA-3′), *BMI1* (5′-CTGATGTGTGTGCTTTGTGG-3′ and 5′-TGGTCTCCAGGTAACGAACA-3′), *PROM1 (CD133)* (5′-CAGTCTGACCAGCGTGAAAA-3′ and 5′-GGATTGATAGCCCTGTTGGA-3′), or *GAPDH* (5′-CATGACCACAGTCCATGCCAT-3′ and 5′-AAGGCCATGCCAGTGAGCTTC-3′) with an annealing temperature of 61 °C.

### Cell proliferation assay

Cell proliferation/viability was determined by cell counting or colorimetric WST assay (Ez-Cytox; Dogenbio, Seoul, Korea). Briefly, cells were seeded into 6-well plates at a density of 5 × 10^4^ cells/well and incubated for 48 or 72 h. Cells were harvested and counted in each well. Cells were seeded in 96-well plates at a density of 5 × 10^3^ cells/well, incubated for 24 h, and further incubated for 72 h in the presence of compound or vehicle. Thereafter, cells were incubated with WST reagent (one-tenth of the medium volume) and the amount of formazan dye formed was determined by measuring absorbance at 450 nm using a spectrophotometric microplate reader (BMG LABTECH GmbH, Ortenberg, Germany).

### Cell survival analysis and anoikis assay

To determine cell survival under suspension culture conditions, cells (1.5 × 10^4^) were seeded into 96-well plates with an Ultra-Low Attachment Surface (Corning, NY), and then incubated for up to 7 days in the absence of serum. Cell viability was determined using the colorimetric WST assay as described above. To induce anoikis, cells (5 × 10^5^) were seeded into 6-well plates with an Ultra-Low Attachment Surface (Corning) for 48 or 72 h. Cells were then stained with 5 μl of annexin V and 5 μl of propidium iodide (PI) per 1 × 10^5^ cells for 15 min at 25 °C in the dark, and the percentage of apoptotic cells was analyzed by flow cytometry.

### Anchorage-independent soft agar assay

Cells (3 × 10^3^) were seeded in 6-well tissue culture plates in 0.3% agar (Sigma) over a 0.6% agar feeder layer. Cells were allowed to grow at 37 °C in 5% CO_2_ for 14 days, and then the colonies in each well were counted.

### Cell adhesion assay

Cells were seeded into 96-well MaxiSorp plates (NUNC, Roskilde, Denmark) coated with 100 μl of fibronectin (10 μg/ml). After 20, 60, or 180 min incubation at 37 °C in 5% CO_2_, adherent cells were fixed with 10% formalin and stained with 2% crystal violet. The number of adherent cells was counted in five representative (**×** 100) fields per well.

### Sphere formation assay (Cancer stem cell spheroid assay)

To induce sphere formation, cells were dissociated to single cells and seeded at a density of 200 cells/well into 96-well plates with an Ultra-Low Attachment Surface to prevent the cells from attaching to the surface. Culture medium was serum-free DMEM/F12 containing 20 ng/ml of EGF (Peprotech, Seoul, Korea), 10 ng/ml of bFGF (Peprotech), and 2% B27 supplement (Thermo Fisher Scientific). Cells were incubated for 7 or 10 days at 37 °C/5% CO_2_. The number of spheroids > 25 or 75 μm in diameter was counted.

### Aldehyde dehydrogenase (ALDH) assay

Cell populations with high ALDH enzymatic activity were identified using the ALDEFLUOR kit (Stem Cell Technologies, Vancouver, Canada). Briefly, 1 × 10^5^ cells were resuspended in ALDEFLUOR assay buffer containing ALDH substrate. As a negative control, an aliquot of ALDEFLUOR-exposed cells was immediately quenched with an ALDH inhibitor, diethylaminobenzaldehyde (DEAB). After 30 min of incubation at 37 °C, the cells were analyzed by flow cytometry.

### Promoter reporter assay

For transfection, cells were seeded into 6-well plates at a density of 2 × 10^5^ cells/well and incubated for 24 h. Cells were then transfected with 2 μg of reporter plasmids using Lipofectamine 2000. At 48 h post-transfection, firefly luciferase activity was measured using a Dual-luciferase reporter assay system (Promega, Southampton, UK). Transfection efficiency was normalized by measuring *Renilla* luciferase activity, encoded by the co-transfected *Renilla* luciferase vector (pRL-TK). For siRNA transfection, cells were co-transfected with siRNA and plasmid for 48 h. The *SOX2* promoter region (− 2546/+ 544) was obtained by PCR with primers (5′-CGTTGGTACCCTAACCTCCCACTTACCTC-3′ and 5′-TCCAAAGCTTGCTGTTTTTCTGGTTGCCGCC-3′), using genomic DNA from HEK293E cells, and then subcloned into the pGL3basic vector (Promega). The *SOX2* promoter (− 484/+ 537) construct in pGL3basic was kindly provided by Addgene (#101761). The mutant *SOX2* promoter reporter construct (ΔE-box) was generated by using the QuikChange site-directed mutagenesis kit (Stratagene, La Jolla, CA). Mutagenesis primers were as follows: 5′-CCCTGACAGCCCCCGTACCATGGATGGTTGTCTA-3′ and its complementary strand.

### Co-immunoprecipitation

HEK293E cells were transfected with a vector expressing SLUG using Lipofectamine 2000. Two days after transfection, cells were lysed with co-immunoprecipitation buffer (10 mM HEPES (pH 8.0), 150 mM NaCl, 0.1 mM EDTA, 20% glycerol, 0.2% NP-40) supplemented with protease inhibitor (Complete, Roche). Lysates were centrifuged for 20 min at 10000 *g* and the resultant supernatant was precleared by incubation with TrueBlot Anti-Mouse Ig IP beads (Rockland Immunochemicals, Inc., Gilbertsville, PA) for 1 h at 4 °C. The precleared supernatant was immunoprecipitated using anti-SOX2 (Santa Cruz Biotechnology, sc-365,823) for 1 h at 4 °C. The protein complexes were collected by incubation with TrueBlot Anti-Mouse Ig IP beads for 16 h at 4 °C, and then washed three times with co-immunoprecipitation buffer. The protein complexes were eluted by boiling in SDS sample buffer and analyzed by immunoblotting with anti-SLUG and anti-SOX2 antibodies.

### Chromatin immunoprecipitation (ChIP)

ChIP assays were performed according to the instructions of the EZ-Magna ChIP Assay Kit (Millipore). Briefly, PC3 cells were transfected with a vector expressing TWIST1 for 48 h, and the equivalent of 1 × 10^6^ cells was used per ChIP reaction with mouse anti-TWIST1. As a control antibody, normal mouse IgG was used. Immunoprecipitated and input DNA were analyzed by PCR with primers specific for the *SOX2* promoter (5′- GCGCTGATTGGTCGCTAGA-3′ and 5′-TCTCTGCCTTGACAACTCCTG-3′ for − 62/+ 45 and 5′-TCCAGAAATACGAGTTGGACA-3′ and 5′-TGCAGGGTACTTAAATGAGG-3′ for − 450/− 345).

### In vivo early metastasis (CTC survival analysis) and xenograft models

All animal procedures were performed in accordance with the guidelines of the Animal Care Committee at the Korea Research Institute of Bioscience and Biotechnology (KRIBB) and with approval of the bioethics committee of the KRIBB (KRIBB-AEC-19098 and KRIBB-AEC-21109). Nude mice (BALB/c-nude, 5-week-old females) were purchased from Nara Biotech (Seoul, Korea). PC3 stable cells (TMPRSS4-overexpressing cells and vector-transfectants; 5 × 10^6^) were injected via the tail vein into nude mice (*n* = 4 per group). Twenty-four hours after injection, mice were sacrificed, the lungs were immediately removed, and total DNA was extracted as previously described [29–31]. Human tumor cell contents present in mouse lungs were determined using real-time qPCR analysis of human prostaglandin E receptor 2 (*PTGER2*) genomic DNA to quantify survival and seeding/arrest of CTCs, as previously reported [29]. qPCR was performed in triplicate using 1 μg of total genomic DNA as a template and *PTGER2*-specific primer set (5′-TACCTGCAGCTGTACGCCAC-3′, 5′-GCCAGGAGAATGAGGTGGTC-3′, and a human *PTGER2*-specific probe FAM 5′-TGCTGCTTCTCATTGTCTCG-3′ TAMRA). A standard curve was generated by qPCR using genomic DNA extracted from PC3 cells and nude mouse lungs (serial dilutions of mouse-only, human-only, or mixed (human + mouse) samples of known DNA concentrations were included as samples). A standard curve with the equation of the linear trend line was developed by plotting the mean Threshold Cycle (*Ct*) values (y-axis) vs. the log of the amount of human genomic DNA (x-axis). The amounts of human genomic DNA initially present in the qPCR reaction tube were extrapolated from the standard curve.

22Rv1 (5 × 10^6^) cells were subcutaneously injected into the flank of nude mice. When the tumor volume reached approximately 80 mm^3^, the mice were randomly grouped (*n* = 6 per group). A mixture of 10 μg TMPRSS4-specific or scrambled shRNA vector and in vivo-jetPEI transfection reagent (Polyplus, New York, NY) was intratumorally injected into mice at an interval of 2 or 3 days (total of 10 times). Tumor volumes were measured and calculated using the formula (**a** × **b**^2^) × 1/2, where **a** was the width at the widest point of the tumor and **b** was the maximal width perpendicular to **a**.

### Analysis of The Cancer Genome Atlas (TCGA) data

cBioPortal (www.cbioportal.org) [32, 33] was used to analyze TCGA-generated human prostate adenocarcinoma (TCGA, Firehose Legacy, and MSKCC, Cancer Cell 2010, studies) [34] and Cancer Cell Line Encyclopedia (CCLE) (Broad, 2019) [35] data. All samples where mRNA expression profiles are available were included in our analysis. Spearman’s correlation coefficient (rho) and *P*-value were calculated using the cBioPortal webpage.

### Statistical analysis

Statistical analyses were performed using Student’s *t*-test, one-way ANOVA (with GraphPad Prism 8 (GraphPad Software, CA)), and Spearman’s test (for correlation analysis). A value of *P* < 0.05 was considered statistically significant.

## Results

### TMPRSS4 promotes anchorage-independent growth of prostate cancer cells

In previous work, we observed that high expression of *TMPRSS4* in prostate cancer patients was significantly correlated with reduced disease-free survival. We also observed that TMPRSS4 not only promoted invasion and EMT, but also increased the growth of primary tumors [22], indicating greater tumorigenicity. Because tumorigenicity reflects tumor-initiating capacity, we investigated whether TMPRSS4 in prostate cancer cells promotes characteristic traits of tumor-initiating/cancer stem cells.

**Fig. 1:**
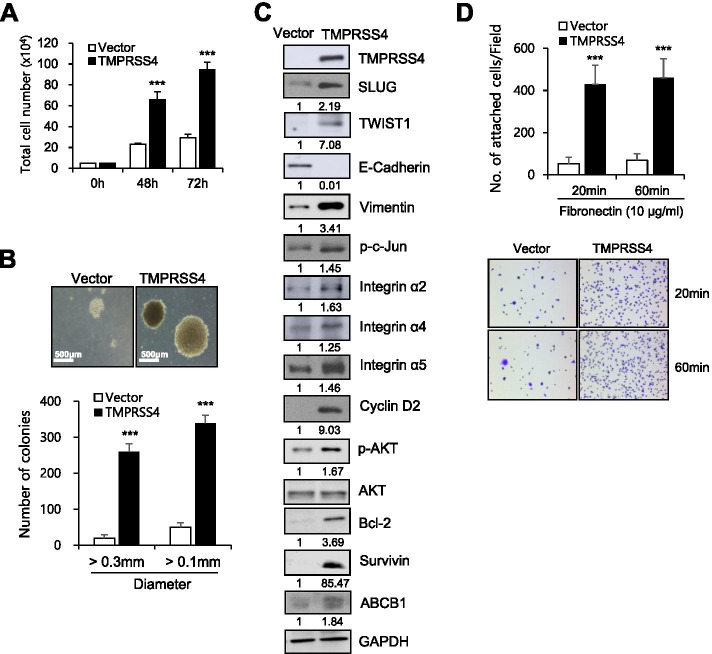
TMPRSS4 promotes anchorage-independent growth of prostate cancer cells. **A** PC3 cells were stably transfected with TMPRSS4 expression vector or empty vector. Cells were seeded in 6-well plates at a density of 5 × 10^4^ cells/well and incubated for 48 or 72 h. Cells were counted to measure proliferation. **B** Soft agar anchorage-independent growth assay. Transfected PC3 cells were seeded in triplicates in 6-well plates containing semi-solid agar at a density of 3 × 10^3^ cells/well and allowed to grow for 14 days. Representative images are shown. Total colonies (> 0.1 mm) and colonies with diameter > 0.3 mm were counted. Bar, 500 μm. **C** Transfected PC3 cells were incubated for 48 h, and lysed for immunoblot analysis. Anti-myc antibody was used to detect myc-tagged TMPRSS4. Densitometric quantification was performed on the immunoblots using GAPDH as a loading control except that phospho-AKT was normalized against total AKT. The mean relative density from three independent experiments is shown under the immunoblots. **D** Cell adhesion assay. Transfected PC3 cells were plated onto fibronectin-coated plates for 20 or 60 min. The number of adherent cells was counted in five representative fields (× 200). Values represent mean ± standard deviation (SD). ***P < 0.001

First, we characterized stable TMPRSS4-overexpressing PC3 prostate cancer cell lines. Consistent with our previous observation that in vivo tumor growth was increased by 5-fold in mouse xenografts consisting of TMPRSS4-overexpressing cells relative to those consisting of vector transfectants [22], TMPRSS4-overexpressing PC3 cells exhibited elevated proliferative activity compared with vector-transfectants in vitro (Fig. 1A). A soft agar assay revealed that TMPRSS4-overexpressing cells grew more and larger colonies over 14 days than control cells (Fig. 1B), indicating that anchorage-independent growth of cells was increased by TMPRSS4 overexpression.

Immunoblot analysis revealed that phosphorylation of c-Jun and expression of SLUG, TWIST1, vimentin, and cyclin D2 were elevated, whereas E-cadherin was reduced, in TMPRSS4-overexpressing PC3 stable cells relative to vector-transfectants (Fig. 1C). Other EMT-transcription factors such as Snail, ZEB1, and ZEB2 were not induced by TMPRSS4 in PC3 cells (data not shown). TMPRSS4 overexpression also increased phosphorylation of AKT, expression of Bcl-2 and survivin, and the levels of integrins α5, α4, and α2 (Fig. 1C). Cell adhesion to fibronection was stronger in TMPRSS4-overexpressing cells than in vector-transfectants (Fig. 1D), possibly due to higher levels of integrin α5 or α4 expression, which may contribute to cell survival. In accordance with this observation, adhesion to fibronectin of PC3 cells resulted in upregulation of survivin and protection from TNFα-induced apoptosis [36].

### TMPRSS4 confers resistance to anoikis and drugs in prostate cancer cells

**Fig. 2:**
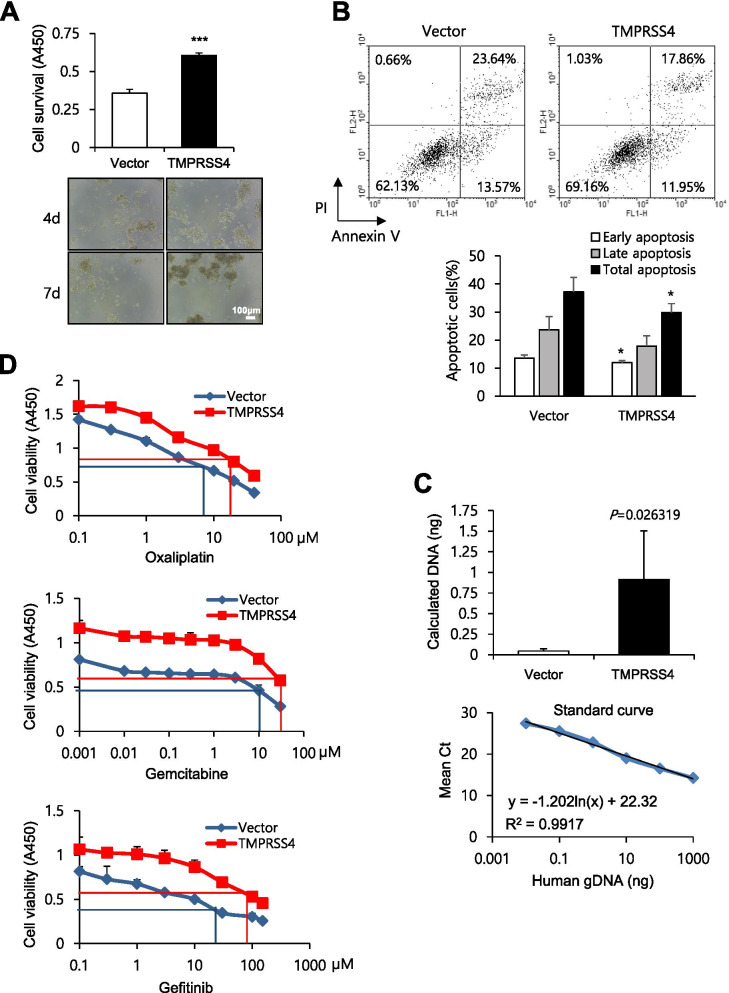
TMPRSS4 promotes resistance to anoikis and drugs. **A** To induce anoikis, transfected PC3 cells were seeded in 96-well plates with an Ultra-Low Attachment Surface at a density of 1.5 × 10^4^ cells/well and grown for up to 7 days. Cell viability was determined by colorimetric WST assay. **B** Transfected PC3 cells were incubated for 48 h with 0.5% bovine serum albumin under suspension culture conditions, and then stained with annexin V and PI for flow cytometry. The proportion of stained cells was determined to calculate the percentage of apoptotic cells. **C** Transfected PC3 cells (5 × 10^6^ cells/mouse) were intravenously injected into nude mice (*n* = 4). Twenty-four hours after injection, lungs were removed to extract total DNA. Real-time qPCR analysis was performed on human *PTGER2* with total DNA extracted from lungs. The amounts of human genomic DNA initially present in the qPCR reaction tube were extrapolated (Upper) from the standard curve generated by real-time qPCR performed on human total DNA extracted from PC3 parental cells mixed with mouse total DNA from lungs of nude mice (Lower). **D** Transfected PC3 cells were treated with anti-cancer drugs for 72 h. Cell viability was determined by colorimetric WST assay. DMSO (0.1%) was used as a vehicle. Values represent mean ± SD. *P < 0.05; ***P < 0.001

We next examined the survival of TMPRSS4-overexpressing cells under suspension culture conditions. When cells were incubated in the absence of serum in low-attachment plates for up to 7 days to induce anoikis, survival of TMPRSS4-overexpressing cells was 69% higher than that of vector-transfectants (Fig. 2A). Flow cytometric analysis also showed that the rate of apoptosis was lower in TMPRSS4-overexpressing cells than in vector-transfectants (Fig. 2B).

Furthermore, we investigated whether TMPRSS4 promotes survival and subsequent seeding of CTCs in vivo using an early metastasis model. TMPRSS4-overexpressing PC3 cells or vector-transfectants were intravenously injected into nude mice. Twenty-four hours after injection, mice were sacrificed and total DNA was extracted from the lungs. The tumor cell content in the lungs was determined by real-time qPCR analysis to detect human *PTGER2* genomic DNA. The amount of human genomic DNA was significantly higher in the lungs of mice injected with TMPRSS4-overexpressing cells than in the lungs of mice injected with vector-transfectants (Fig. 2C), suggesting that TMPRSS4 promotes CTC survival, thereby supporting early metastasis.

In addition, when PC3 cells transfected with TMPRSS4 expression vector or empty vector were treated with oxaliplatin, gemcitabine, or gefitinib for 72 h, TMPRSS4-overexpressing PC3 cells exhibited lower drug sensitivity than vector-transfectants (IC_50_ = 20 μM vs. 8 μM for oxaliplatin, IC_50_ = 30 μM vs. 10.5 μM for gemcitabine, IC_50_ = 80 μM vs. 20 μM for gefitinib) (Fig. 2D). Consistent with this, TMPRSS4 overexpression increased expression of MDR1/ABCB1 (Fig. 1C).

### TMPRSS4 promotes tumorsphere formation and upregulates stemness-related factors

**Fig. 3:**
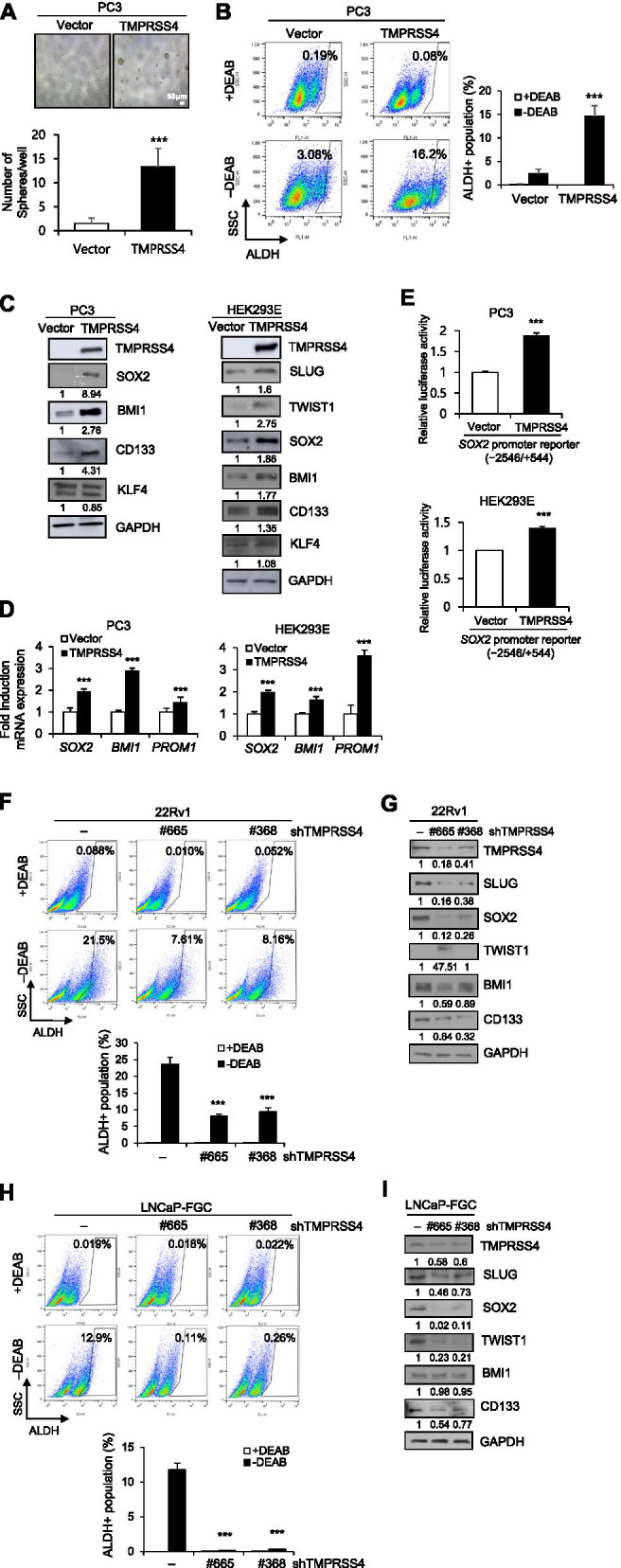
TMPRSS4 promotes tumorsphere formation and upregulates stemness-related factors. **A** Tumorsphere formation assay. Transfected PC3 cells were dissociated to single cells and seeded at a density of 200 cells/ml in 96-well plates under suspension culture conditions in the presence of 20 ng/ml EGF, 10 ng/ml bFGF, and 2% B27 supplement. The cells were incubated for 7 days. Spheroids with diameter > 75 μm were counted. Bar, 50 μm. **B** ALDH assay. Transfected PC3 cells were incubated with ALDH substrate for 30 min, and then analyzed using flow cytometry. Cells were quenched with DEAB, an ALDH inhibitor, as a negative control. **C** PC3 and HEK293E cells transfected with TMPRSS4 expression vector were lysed for immunoblot analysis. Anti-myc antibody was used to detect myc-tagged TMPRSS4. **D** Transfected cells were lysed and subjected to real-time qPCR analysis. **E** Cells were co-transfected with a TMPRSS4 expression vector and a *SOX2* promoter (− 2546/+ 544) reporter construct in the pGL3 vector. Firefly luciferase activity, representing *SOX2* promoter activity, was measured after 48 h and normalized against *Renilla* luciferase activity. **F–I** 22Rv1 and LNCaP clone FGC cells were transfected with TMPRSS4-specific shRNA vectors for 48 h. Transfected cells were subjected to ALDH assay (**F, H**) or lysed for immunoblot analysis (**G, I**). Densitometric quantification was performed on the immunoblots using GAPDH as a loading control. The mean relative density from three independent experiments is shown under the immunoblots (**C, G, I**). Values represent mean ± SD. ***P < 0.001

To determine whether TMPRSS4 promotes tumorsphere formation (a reflection of tumor-initiating capacity), cells were incubated in DMEM/F12 containing 20 ng/ml EGF, 10 ng/ml bFGF, and 2% B27 supplement under suspension culture conditions. TMPRSS4-overexpressing PC3 cells formed more tumorspheres over 7 days than vector-transfectants (Fig. 3A). We also analyzed aldehyde dehydrogenase (ALDH) activity in TMPRSS4-overexpressing cells, as elevated ALDH activity is associated with CSC features and poor prognosis in several cancers, including prostate cancer [37]. The proportion of ALDH+ cells was substantially higher in TMPRSS4-overexpressing PC3 cells than in vector-transfectants (Fig. 3B). Immunoblot analysis revealed that TMPRSS4 upregulated expression of the stemness-related factors SOX2, BMI1, and CD133 in PC3 cells, whereas KLF4 was not increased by TMPRSS4 overexpression. Similarly, in HEK293E cells, TMPRSS4 upregulated SOX2, BMI1, and CD133, as well as SLUG and TWIST1 (Fig. 3C). On the other hand, DU145 cells had a higher proportion of ALDH+ cells following TMPRSS4 overexpression (Supplementary Fig. S1), although DU145 cells failed to form spheres.

Real-time qPCR analysis revealed that *SOX2*, *BMI1*, and *PROM1 (CD133)* mRNA expression in PC3 and HEK293E cells was significantly increased by TMPRSS4 (Fig. 3D). A reporter assay showed that TMPRSS4 induced 1.88- and 1.40-fold increases in *SOX2* promoter (− 2546/+ 544) activity at 48 h post-transfection in PC3 and HEK293E cells, respectively (Fig. 3E).

Endogenous TMPRSS4-expressing 22Rv1 and LNCaP clone FGC prostate cancer cells were transiently transfected with TMPRSS4-specific shRNA vectors. TMPRSS4 suppression significantly reduced ALDH activity (Fig. 3F) and decreased expression of SLUG, SOX2, BMI1, and CD133 in 22Rv1 cells, although TWIST1 was aberrantly upregulated by TMPRSS4 suppression (Fig. 3G). TMPRSS4 suppression significantly reduced ALDH activity (Fig. 3H) and decreased expression of SLUG, SOX2, TWIST1, and CD133 in LNCaP clone FGC cells (Fig. 3I). TMPRSS4 suppression significantly reduced tumorsphere formation, cell adhesion, and cell survival, and significantly elevated the rate of apoptosis in 22Rv1 and LNCaP clone FGC cells (Supplementary Fig. S2). To evaluate the effect of TMPRSS4 suppression in vivo, 22Rv1 cells were injected subcutaneously into the flanks of nude mice and then the control vector or TMPRSS4-specific shRNA vector was intratumorally injected. TMPRSS4 suppression in the 22Rv1 xenograft model significantly decreased tumor growth (Supplementary Fig. S2E). Similarly, suppression of endogenous TMPRSS4 reduced ALDH activity in HT-29 and HCT-116 colon cancer cells, and this was accompanied by downregulation of SLUG, TWIST1, and SOX2, but not BMI1 or CD133 (Supplementary Fig. S3).

### SLUG and TWIST1 are required for TMPRSS4-mediated promotion of CSC features

Next, we explored the molecular mechanisms by which TMPRSS4 promotes cancer stem–like properties. EMT-inducing transcription factors play important roles in stemness [8, 9]. Therefore, we predicted that SLUG or TWIST1 could contribute to TMPRSS4-mediated induction of cancer stem–like properties. We previously observed that TMPRSS4 activates the transcription factors AP-1 and SP1 [26], and upregulates SLUG expression in an AP-1-dependent manner [22]. To explore whether AP-1 or SP1 is involved in TMPRSS4-induced upregulation of SLUG and TWIST1, PC3 cells were co-transfected with TMPRSS4 expression vector and siRNA specific to c-Jun or SP1. Real-time qPCR and immunoblot analyses showed that TMPRSS4-induced expression of SLUG and TWIST1 decreased following suppression of c-Jun or SP1. This was accompanied by reduction of SOX2 (Supplementary Fig. S4A and B). In addition, c-Jun and SP1 overexpression upregulated SLUG and TWIST1 expression in PC3 cells (Supplementary Fig. S4C). Consistently, several reports demonstrated that AP-1 and SP1 activities are involved in SLUG [38, 39] and TWIST1 [40–42] expression.

**Fig. 4:**
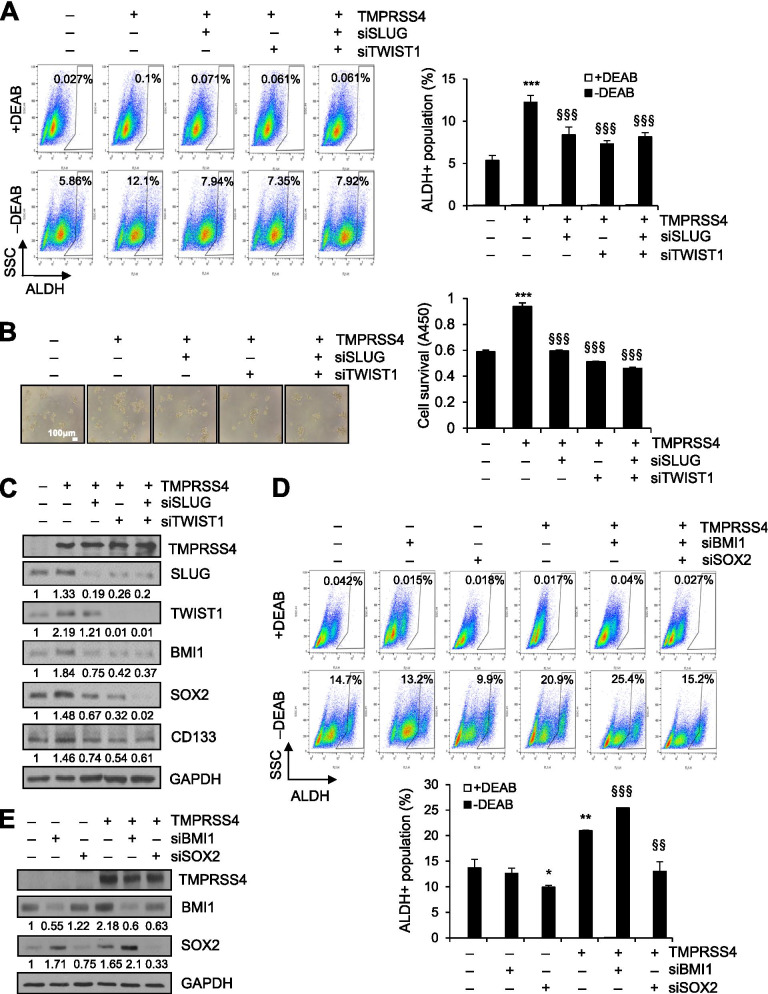
SLUG and TWIST1 are required for TMPRSS4-mediated ALDH activity and anoikis resistance. **A–C** PC3 cells were co-transfected with a TMPRSS4 expression vector and siRNA specific to SLUG or TWIST1 for 48 h. **A** Transfected cells were harvested and then subjected to ALDH assay. **B** Transfected cells were incubated under suspension culture conditions and then cell viability was determined by colorimetric WST assay. **C** Transfected cells were lysed for immunoblot analysis. **D, E** PC3 cells were co-transfected with TMPRSS4 expression vector and siRNA specific to BMI1 or SOX2 for 48 h. Transfected cells were subjected to ALDH assay (**D**) or lysed for immunoblot analysis (**E**). Anti-myc antibody was used to detect myc-tagged TMPRSS4. Densitometric quantification was performed on the immunoblots using GAPDH as a loading control. The mean relative density from three independent experiments is shown under the immunoblots (**C, E**). Values represent mean ± SD. *P < 0.05; **P < 0.01; ***P < 0.001 compared with vector + control siRNA; ^§§^*P* < 0.01; ^§§§^*P* < 0.001 compared with TMPRSS4 + control siRNA

To investigate the role of SLUG and TWIST1 in TMPRSS4-mediated ALDH activation and anoikis resistance, PC3 cells were co-transfected with TMPRSS4 expression vector and siRNA against SLUG or TWIST1 for 48 h, and then subjected to ALDH activity and anoikis assays. TMPRSS4-induced ALDH activity (Fig. 4A) and TMPRSS4-induced cell survival under suspension conditions (Fig. 4B) were significantly decreased by suppression of either SLUG or TWIST1. Immunoblot analysis revealed that TMPRSS4-induced expression of SOX2, BMI1, and CD133 (relatively moderately) was reduced, following suppression of SLUG and TWIST1 (Fig. 4C). Interestingly, suppression of SLUG decreased TWIST1 expression, and depletion of TWIST1 decreased SLUG expression, indicating reciprocal regulation between SLUG and TWIST1 (Fig. 4C). Furthermore, ALDH activation by TMPRSS4 was decreased by depletion of SOX2, whereas ALDH activation by TMPRSS4 was moderately increased by depletion of BMI1 (Fig. 4D), indicating that SOX2 plays a critical role in TMPRSS4-induced CSC traits. Consistent with this, suppression of BMI1 resulted in elevated SOX2 expression (Fig. 4E). In addition, suppression of SOX2 significantly decreased TMPRSS4-mediated tumorsphere formation and cell survival under suspension conditions and significantly attenuated the TMPRSS4-induced decrease in the rate of apoptosis (Supplementary Fig. S5), confirming that SOX2 plays a role in TMPRSS4-induced CSC traits. Together, these results suggest that SLUG and TWIST1 are required for TMPRSS4-induced SOX2 upregulation and subsequent acquisition of CSC features.

### SLUG upregulates SOX2 expression at the level of protein stability, promoting acquisition of CSC features

**Fig. 5:**
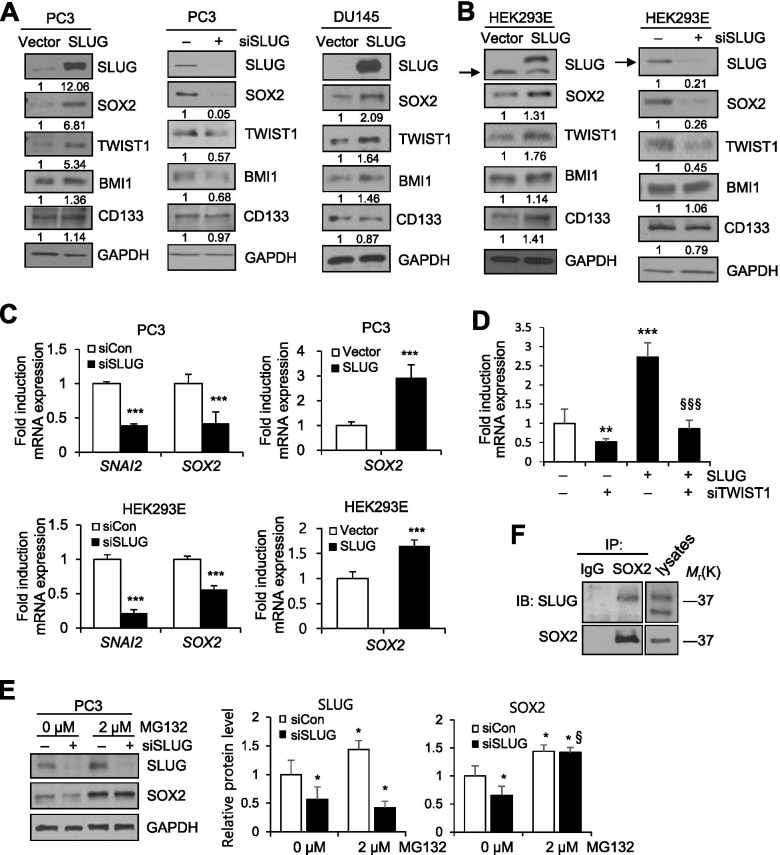
SLUG stabilizes SOX2, leading to ALDH activation and anoikis resistance. **A, B** Cells were transfected with SLUG expression vector or siRNA specific to SLUG for 48 h, and then whole-cell lysates were prepared for immunoblot analysis. Anti-myc antibody was used to detect myc-tagged SLUG (**A** right). Arrow indicates endogenous SLUG (**B**). **C** Cells were transfected for 48 h with a SLUG expression vector or siRNA specific to SLUG, and then lysed for real-time qPCR analysis. Values represent mean ± SD. ***P < 0.001. **D** PC3 cells were co-transfected with SLUG expression vector and siRNA specific to TWIST1 for 48 h, and then lysed for real-time qPCR analysis of *SOX2* expression. Values represent mean ± SD. **P < 0.01; ***P < 0.001 compared with siControl + Vector; ^§§§^*P* < 0.001 compared with siControl + SLUG. **E** PC3 cells were transfected with siRNA for 42 h and then treated with MG132 or vehicle control (0.1% DMSO) for 6 h before lysates preparation for immunoblot analysis. Values represent mean ± SD. *P < 0.05 compared with siControl + DMSO; ^§^*P* < 0.05 compared with siSLUG + DMSO. Densitometric quantification was performed on the immunoblot using GAPDH as a loading control. The mean relative density from three independent experiments is shown (**A, B, E**). **F** Co-immunoprecipitation analysis of the interaction of SLUG with SOX2 in HEK293E cells transfected with SLUG

We next investigated whether and how SLUG modulates SOX2 expression. PC3 cells were transiently transfected with SLUG expression vector or siRNA against SLUG. Immunoblot analysis revealed that SLUG increased expression of SOX2 and TWIST1. SLUG moderately upregulated BMI1 (Fig. 5A left). Conversely, suppression of endogenous SLUG substantially decreased expression of SOX2, TWIST1, and BMI1 but not CD133 (Fig. 5A middle). In addition, in HEK293E cells, SLUG overexpression increased expression of SOX2, TWIST1, and CD133, and depletion of SLUG decreased expression of SOX2, TWIST1, and CD133 (moderately). On the other hand, expression of BMI1 was not substantially affected by SLUG (Fig. 5B). In DU145 cells, SLUG overexpression increased expression of SOX2, TWIST1, and BMI1 but not CD133 (Fig. 5A right), confirming regulation of SOX2 and TWIST1 by SLUG. Meanwhile, TMPRSS4 expression was not apparently changed by SLUG or TWIST1 in PC3, DU145, and HEK293E cells (Supplementary Fig. S6), which normally display little endogenous TMPRSS4 expression, indicating that SLUG and TWIST1 are not involved in regulation of TMPRSS4 expression in our system. On the other hand, SOX2 suppression did not substantially affect expression of SLUG, TWIST1, CD133, or BMI1 (moderately reduced in HEK293E cells), in PC3 and HEK293E cells (Supplementary Fig. S7). Real-time qPCR analysis revealed that *SOX2* mRNA expression was significantly decreased by suppression of SLUG but significantly increased by SLUG overexpression in both PC3 and HEK293E cells (Fig. 5C). However, SLUG did not increase *SOX2* promoter (− 2546/+ 544) activity in PC3 cells (data not shown), indicating that SLUG may not be directly involved in *SOX2* transcription. SLUG-induced *SOX2* mRNA expression was significantly decreased by TWIST1 depletion (Fig. 5D), indicating that TWIST1 is required for SLUG-induced *SOX2* transcription.

TMPRSS4-induced SOX2 expression was reduced by suppression of either SLUG or TWIST1, and the reduction was greater following suppression of both factors (Fig. 4C), indicating that regulation of SOX2 expression by SLUG and TWIST1 may not be completely redundant. To explore the mechanism by which SLUG promotes SOX2 expression, we tested the hypothesis that SLUG-mediated SOX2 upregulation is due in part to an increase in protein stability. PC3 cells were transiently transfected with SLUG-specific siRNA for 42 h and then treated with the proteasome inhibitor MG132 for 6 h before lysis. MG132 substantially reversed the reduction in SOX2 expression mediated by SLUG depletion (Fig. 5E), indicating that SLUG suppression promotes proteasome-dependent degradation of SOX2 (Notably, MG132 increased the basal level of SOX2 to some extent). To determine whether SLUG interacts with SOX2, we performed co-immunoprecipitation studies using lysates from HEK293E cells overexpressing SLUG. SLUG co-precipitated with SOX2 (Fig. 5F). These results suggest that SLUG upregulated SOX2 through binding to SOX2, thereby enhancing its stability.

### TWIST1 upregulates *SOX2* transcription through the E-box element, contributing to stemness

**Fig. 6:**
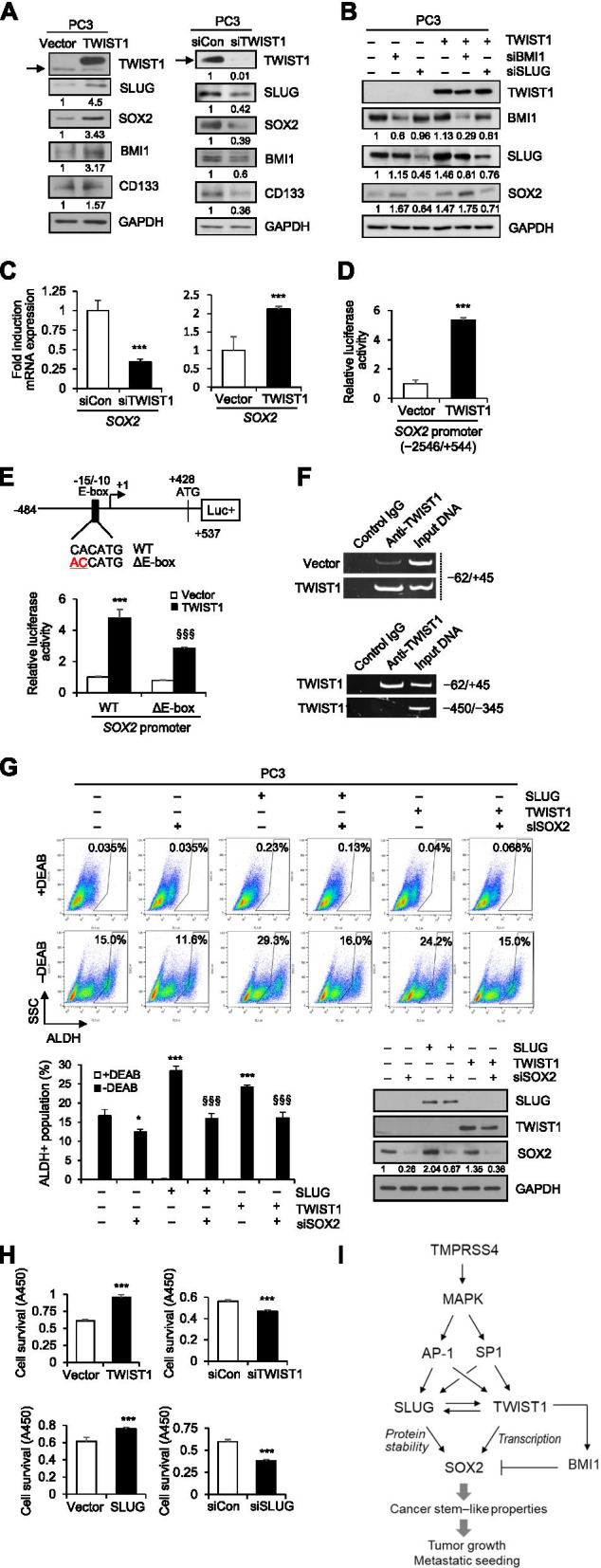
TWIST1 upregulates *SOX2* transcription through the E-box element, contributing to stemness. **A** PC3 cells were transfected for 48 h with TWIST1 expression vector or siRNA specific to TWIST1, and then lysed for immunoblot analysis. Arrow indicates endogenous TWIST1. **B** PC3 cells were co-transfected for 48 h with TWIST1 expression vector and siRNA specific to TWIST1 or BMI1, and then lysed for immunoblot analysis. Anti-flag antibody was used to detect flag-tagged TWIST1. **C** Cells were transfected for 48 h with siRNA specific to TWIST1 or TWIST1-expression vector and then lysed for real-time qPCR analysis. **D** PC3 cells were co-transfected with a TWIST1 expression vector and a *SOX2* promoter (− 2546/+ 544) reporter construct in the pGL3 vector. Values represent mean ± SD. ***P < 0.001. **E** An E-box site mutant reporter construct was generated from the *SOX2* promoter (− 484/+ 537) reporter construct in the pGL3 vector and used in reporter assay. Values represent mean ± SD. ***P < 0.001 compared with vector + WT; ^§§§^*P* < 0.001 compared with TWIST1 + WT. **F** ChIP analysis of the interaction of TWIST1 with the *SOX2* promoter. Upper, Chromatin fragments from PC3 cells transfected for 48 h with a TWIST1 expression vector or an empty vector were immunoprecipitated with control mouse IgG or anti-TWIST1 and analyzed by PCR using primers specific for the *SOX2* promoter (− 62/+ 45). Lower, ChIP assay using PC3 cells transfected with TMPRSS4 expression vector for 48 h. An irrelevant region (− 450/− 345) was analyzed in parallel. The input control (1%) is in lane 3. **G** PC3 cells were transfected with a SLUG or TWIST1 expression vector and siRNA specific to SOX2 for 48 h and then subjected to ALDH assay and immunoblot analysis. Anti-flag antibody was used to detect flag-tagged TWIST1. Values represent mean ± SD. *P < 0.05; ***P < 0.001 compared with vector + control siRNA; ^§§§^*P* < 0.001 compared with SLUG or TWIST1 + control siRNA. Densitometric quantification was performed on the immunoblot using GAPDH as a loading control. The mean relative density from three independent experiments is shown under the immunoblots (**A, B, G**). **H** PC3 cells were transfected with a SLUG or TWIST1 expression vector or siRNA specific to SLUG or TWIST1 for 48 h. Transfected cells were incubated under suspension culture conditions. Cell viability was determined by colorimetric WST assay. Values represent mean ± SD. ***P < 0.001. **I** A schematic representation illustrating the pathways underlying TMPRSS4-induced cancer stem–like properties in human cancer cells. We previously reported that TMPRSS4 activates the transcription factors AP-1 and SP1 in a manner dependent on MAPKs including JNK and ERK1/2 [26]. Our previous [22] and present studies show that TMPRSS4 upregulates SLUG and TWIST1 in an AP-1- and SP1-dependent manner. This study demonstrates that TMPRSS4 upregulates SOX2 at the transcriptional and protein stability levels through TWIST1 and SLUG, respectively, leading to acquisition of cancer stem–like features, including ALDH activation, thereby contributing to tumor growth and metastatic seeding. Of note, expression levels of SLUG and TWIST1 are interdependent. BMI1 suppression enhances SOX2 expression, indicating that BMI1 negatively regulates SOX2 expression in our system

We next explored whether and how TWIST1 modulates SOX2 expression. PC3 cells were transiently transfected with TWIST1 expression vector or siRNA against TWIST1. Immunoblot analysis revealed that TWIST1 substantially increased expression of SOX2, SLUG, BMI1, and CD133 (Fig. 6A left). Consistently, TWIST1 upregulates BMI1 in head and neck squamous cancer cells [43] and SLUG in human mammary epithelial cells [44]. Suppression of endogenous TWIST1 substantially decreased expression of SOX2, SLUG, BMI1, and CD133 in PC3 cells (Fig. 6A right). Furthermore, induction of SOX2 by TWIST1 was decreased by knockdown of SLUG, whereas SOX2 expression was increased by suppression of BMI1 (Fig. 6B), consistent with Fig. 4E, indicating that SLUG plays a role in TWIST1-induced SOX2. These observations confirm the presence of bidirectional regulation between SLUG and TWIST1 (see Figs. 4C and 5A).

Real-time qPCR analysis revealed that TWIST1 suppression reduced *SOX2* mRNA levels in PC3 cells. Conversely, TWIST1 overexpression enhanced *SOX2* mRNA levels (Fig. 6C). A reporter assay showed that TWIST1 induced 5.3- and 4.8-fold increases in activity of *SOX2* promoter regions (− 2546/+ 544) and (− 484/+ 537), respectively (Fig. 6D and E). The region (− 484/+ 537) contains a putative E-box element (− 15/− 10). Mutation of this element significantly decreased the TWIST1-induced activation of the *SOX2* promoter (Fig. 6E). The interaction of TWIST1 with the *SOX2* promoter was examined in TWIST1-transfected PC3 cells by chromatin immunoprecipitation (ChIP). Chromatin fragments containing the *SOX2* promoter (− 62/+ 45) were efficiently pulled down by an anti-TWIST1 antibody from TWIST1-transfected cells compared with cells transfected with the empty vector (Fig. 6F). Immunoprecipitation with anti-TWIST1 antibody specifically pulled down chromatin fragments containing the *SOX2* promoter (− 62/+ 45) but did not pull down an irrelevant region (− 450/− 345), indicating that TWIST1 upregulated *SOX2* transcription via direct binding with the proximal E-box element.

Elevated expression of SLUG and TWIST1 significantly increased ALDH activity in PC3 cells, and this activity was significantly decreased by suppression of SOX2 (Fig. 6G), indicating that SOX2 is a critical mediator of SLUG- and TWIST1-mediated cancer stem–like properties. SLUG and TWIST1 also significantly modulated cell survival under suspension conditions (Fig. 6H).

Together, these results indicate that TWIST1 and SLUG upregulate SOX2 expression at the level of transcription and protein stability, respectively, leading to acquisition of CSC characteristics. These results also suggest the presence of bidirectional regulation between SLUG and TWIST1.

### Clinical significance of *TMPRSS4*, *SOX2*, *SNAI2*, and *TWIST1* expression in prostate cancer patients

**Fig. 7:**
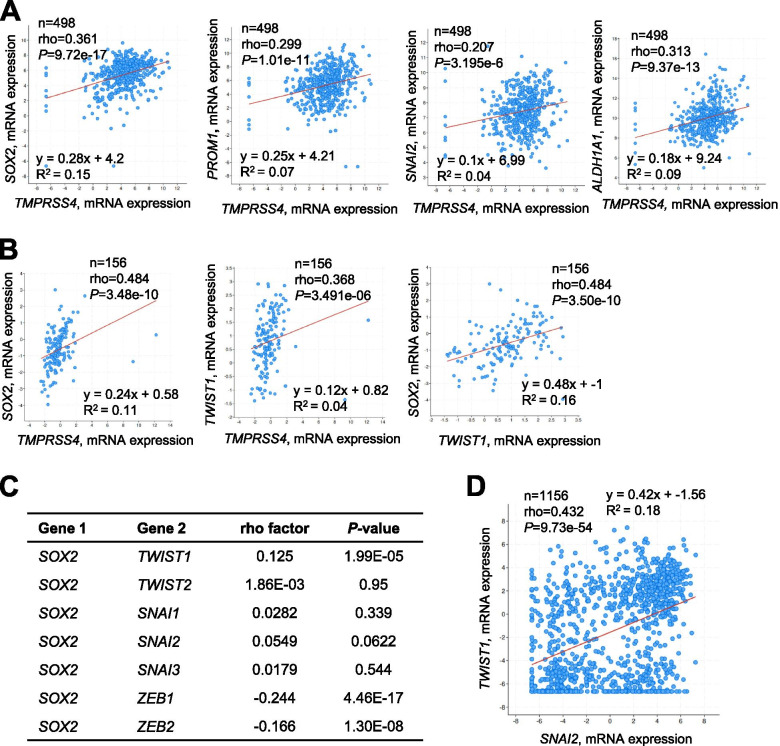
Clinical significance of *TMPRSS4*, *SNAI2*, *TWIST1*, and *SOX2* expression in prostate cancer patients. **A** Scatter plots of *TMPRSS4* mRNA expression (x-axis) vs. *SOX2*, *PROM1* (CD133), *SNAI2*, or *ALDH1A1* mRNA expression (y-axis) from prostate adenocarcinoma data (TCGA, Firehose Legacy). **B** Scatter plots of *TMPRSS4* mRNA expression (x-axis) vs. *SOX2* or *TWIST1* mRNA expression (y-axis) from prostate adenocarcinoma data (MSKCC, Cancer Cell 2010). A scatter plot of *TWIST1* mRNA expression (x-axis) vs. *SOX2* mRNA expression (y-axis) is also shown. **C** Correlation analysis between expression levels of *SOX2* and several EMT-inducing transcription factors (*n* = 1156) from CCLE data (Broad, 2019). **D** A scatter plot of *SNAI2* mRNA expression (x-axis) vs. *TWIST1* mRNA expression (y-axis) from CCLE data (Broad, 2019). Correlations were statistically analyzed using the Spearman test. Equations were automatically generated using the cBioPortal webpage tool

To determine whether TMPRSS4 expression correlates with SLUG, TWIST1, and stemness-related factors expression in human cancers, we analyzed TCGA-generated prostate adenocarcinoma data (TCGA, Firehose Legacy and TCGA, Cancer Cell 2010 studies) [34]. Correlation was analyzed by calculating Spearman’s correlation coefficient (rho). Analysis of the Firehose Legacy dataset showed that *TMPRSS4* expression was significantly correlated with expression of *SOX2* (rho = 0.361, *P* = 9.72e-17), *ALDH1A1* (rho = 0.313, *P* = 9.37e-13), *PROM1* (rho = 0.299, *P* = 1.01e-11), and *SNAI2* (rho = 0.207, *P* = 3.195e-6) (Fig. 7A), but not with expression of *KLF4* (rho = 0.0614, *P* = 0.171) or *BMI1* (rho = 0.0421, *P* = 0.349) (graph not shown). In addition, analysis of the Cancer Cell 2010 dataset showed that TMPRSS4 expression was significantly correlated with expression of *SOX2* (rho = 0.484, *P* = 3.48e-10) and *TWIST1* (rho = 0.368, *P* = 3.491e-06). *TWIST1* expression was significantly correlated with *SOX2* expression (rho = 0.484, *P* = 3.50e-10) (Fig. 7B).

We interrogated the CCLE data (Broad, 2019) [35] to determine the mRNA expression levels of *SOX2* and EMT-inducing transcription factors in various cancer cell lines (*n* = 1156). *SOX2* expression was positively correlated with *TWIST1* expression (rho = 0.125, *P* = 1.99e-05), although the correlation was very weak. However, expression of other EMT-inducing transcription factors such as *TWIST2*, *SNAI1*, *SNAI2*, and *SNAI3* was not significantly correlated with expression of *SOX2*, whereas expression of *ZEB1* and *ZEB2* was negatively correlated with expression of *SOX2* (Fig. 7C). In addition, *SNAI2* expression was positively correlated with expression of *TWIST1* (rho = 0.432, *P* = 9.73e-54) (Fig. 7D).

## Discussion

Elevated expression of TMPRSS4 in various types of cancer, including pancreatic, thyroid, lung, colon, and prostate cancers, is correlated with poor prognosis [19, 21, 45]. Previously, we reported that TMPRSS4 induces EMT and invasion of colon, prostate, and lung cancer cells [23, 26]. We also showed that TMPRSS4 promotes invasion and proliferation of lung and prostate cancer cells through transcription factors AP-1 and SP1 in a manner dependent on MAPKs [22, 26]. In this study, we report that TMPRSS4 confers stem–like properties to prostate cancer cells, mainly through upregulation of SLUG, TWIST1, and SOX2, leading to CTC survival and thereby contributing to metastasis as well as tumorigenicity (Fig. 6I). These results suggest that TMPRSS4 could serve as a novel marker for CSCs in prostate cancer. Our previous [22, 26] and present studies showed that TMPRSS4 contributes to tumor progression through diverse molecular mechanisms, linking the EMT and tumorigenic programs. Further studies should investigate whether TMPRSS4 promotes tumor initiation using transgenic mouse models.

Consistent with our findings, a recent study showed that TMPRSS4 induces cancer stem–like properties in lung cancer cells and correlates with ALDH expression in NSCLC patients [46]. On the other hand, we did not observe TMPRSS4-, SLUG-, or TWIST1-mediated upregulation of SOX2, in lung cancer cells (data not shown), implying that TMPRSS4 contributes to CSC functions through various molecular mechanisms depending on the cellular context. On the other hand, to our knowledge, levels of TTSPs other than TMPRSS4 are not correlated with CSC traits at the molecular level, although matriptase, the most studied member of the TTSP family, induces tumorigenesis in mouse models [19]. These studies suggest that TMPRSS4 could serve as a molecular target for anti-cancer therapy, and that inhibition of TMPRSS4 may have potential as a therapeutic strategy to reduce tumor growth and metastasis. Based on the result of TMPRSS4-mediated CSC features, blockade of TMPRSS4 represents a promising approach to overcome drug resistance.

Cancer stem–like features account for the relative aggressiveness of tumors, serving as the driving force of tumorigenesis and the seeds of metastases, and are thus potential prognostic indicators in cancer patients. Expression of a combination of CSC markers or elevated ALDH activity has been used as a CSC biomarker [37, 47]. Reactivation of an embryonic EMT program is commonly accepted as a core component of carcinoma progression. Over the past decade, several lines of evidence have suggested that the roles of EMT-inducing transcription factors in cancer progression are not limited to regulation of cancer cell invasion and dissemination, but instead play multiple pivotal roles, including regulation of CSC properties, cell fate, plasticity, and drug resistance [9]. However, its precise mechanisms remain largely unclear. Certain types of cancer cells, such as breast, colorectal, ovarian, pancreatic and prostate cancers, can acquire tumor-initiating capability after induction of EMT programs [2]. For example, ZEB1 promotes tumorigenicity by repressing stemness-inhibiting microRNAs in pancreatic cancer cells, promoting migration of CSCs [48]. TWIST1 upregulates BMI1, which is essential for promoting EMT and tumor-initiating capability, in head and neck squamous cell carcinoma [43]. In addition, elevated SLUG expression is correlated with overexpression of stem–like genes, including CD133 and BMI1, in basal-type breast tumors [49], and breast tumors overexpressing SLUG display increased proportion of CD44^+^/CD24^−^ CSCs [50], suggesting that the transcriptional programs induced by SLUG promote stemness.

SLUG induces certain mesenchymal genes such as SLUG (autoactivation) [51], ZEB1 [52], and CD147 [53]. In addition, we showed that SLUG induces vimentin [54]. However, its molecular mechanisms was not elucidated whereas it is well known that SLUG, like other EMT-inducing transcription factors, directly represses E-cadherin transcription [5]. In this study, we made the novel observation that SLUG interacts with SOX2 to prevent its proteasomal degradation, thereby stabilizing it. Similarly, SLUG is required for SOX9 stabilization, which promotes CSCs and metastasis in lung cancer [55]. Binding of SLUG likely alters the conformation of SOX2, which interferes with its ubiquitination and subsequent proteasomal degradation. It is also possible that SLUG regulates SOX2 protein stability by regulating certain ubiquitination-related factors.

TWIST1 directly represses *CD24* expression through the E-box element to increase the CD44^+^/CD24^−/low^ cancer stem–like cell population in breast cancer cells [56]. In addition, TWIST1 upregulates transcription of *SNAI2*, *CDH2*, and *BMI1* by recognizing the E-box sequences CATCTG, CATGTG, and CAAGTG within the intron or regulatory regions of the *SNAI2*, *CDH2*, and *BMI1* genes, respectively [43, 44, 57]. Our study shows that TWIST1 can recognize the proximal E-box element CACATG in the *SOX2* promoter, acting as a transcriptional activator, thereby contributing to CSC features. SLUG is an essential mediator of TWIST1-induced EMT in breast cancer cells [44]. We also observed reciprocal regulation between SLUG and TWIST1, which leads to induction of SOX2 and stemness in prostate cancer cells, possibly contributing to the accelerated aggressiveness of the malignancy.

Clinically, we found several associations between expression of *TMPRSS4* and stemness factors, consistent with our experimental data. Analysis of prostate cancer data from TCGA revealed a significant positive correlation between expression of *TMPRSS4* and that of *SNAI2*, *TWIST1*, *SOX2*, *PROM1*, and *ALDH1A1*. We also observed a significant positive correlation between the expression levels of *TWIST1* and *SOX2*. Consistently, analysis of CCLE data revealed that *SOX2* expression was positively correlated with expression of *TWIST1*, but not other EMT-inducing transcription factors. In this study, we demonstrated that TWIST1 directly upregulated *SOX2* transcription, while SLUG enhanced SOX2 protein stability. However, we cannot completely rule out the possibility that TMPRSS4-mediated SOX2 expression is modulated indirectly or via different signaling pathway(s). For example, we previously observed that SLUG induces expression of AXL and subsequent MAPK signaling activities [22], suggesting that SLUG mediates other transcriptional activities, resulting in upregulation of SOX2.

The pluripotency-inducing transcription factor SOX2 is an essential embryonic stem cell gene that is necessary for induced cellular reprogramming. Furthermore, SOX2 is overexpressed and associated with poor prognosis in almost all human cancer types; it is considered to be a key regulator of tumorigenicity, drug resistance, and metastasis in a variety of tumors, including cancers of the ovary, lung, skin, brain, breast, prostate, and pancreas [12, 13]. In prostate cancer, elevated SOX2 expression is associated with poor prognosis and relapse [13]. SOX2 plays a critical role in EGFR-mediated self-renewal of human prostate cancer stem–like cells derived from DU145 cells [58]. SOX2 expression in the primary tumor is significantly associated with lymph node metastasis and highly aggressive neuroendocrine differentiation of prostate cancer [59]. SOX2 and hedgehog signaling together promote prostate cancer cell proliferation and migration and prevent apoptosis [60]. SOX2 promotes antiandrogen resistance through lineage plasticity in prostate cancer [61]. Therefore, SOX2 is an attractive target for cancer therapy; however, considering that SOX2 is a transcription factor, it will be important to identify upstream or downstream regulators that could be drugged. In this study, we showed that TMPRSS4 upregulates SOX2 expression through the EMT-inducing transcription factors TWIST1 and SLUG, suggesting potential role for TMPRSS4/TWIST1–SLUG on acquisition of CSC traits and aggressive malignancy during prostate cancer progression. Targeting TMPRSS4 represents a potential approach to anti-cancer therapy aimed at suppressing tumor growth, metastasis, and drug resistance/relapse by suppressing CSC traits.

## Conclusions

In summary, TMPRSS4 gives prostate cancer cells cancer stem–like features, including ALDH activation, tumorsphere formation, and resistance to anoikis and drugs, thereby contributing to tumor growth and metastatic seeding. The underlying mechanism involves upregulation of SOX2 at the transcriptional and protein stability levels through TWIST1 and SLUG, respectively. Together, these data suggest that the TMPRSS4/TWIST1–SLUG/SOX2 axis could be exploited as a target for anti-cancer therapy.

## Supplementary Information

**Additional file 1: Supplementary Figure S1.** TMPRSS4 promotes ALDH activation in DU145 cells. Transfected DU145 cells were incubated with ALDH substrate for 30 min and then analyzed by flow cytometry. Cells were quenched with DEAB, an ALDH inhibitor, as a negative control. **Supplementary Figure S2.** Effects of TMPRSS4 suppression in 22Rv1 and LNCaP clone FGC cells in vitro and in vivo. (A-D, F-H) Cells were transfected with TMPRSS4-specific shRNA vector (#665) for 48 h. Transfected cells were subjected to tumorsphere formation (A and F), cell survival (B and G), anoikis (C, H), and cell adhesion (D) assays. The number of spheroids > 75 μm (for 22Rv1 cells) or > 25 μm (for LNCaP-FGC cells) in diameter was counted after 10 days. (E) In vivo tumor growth analysis. 22Rv1 (5 × 10^6^) cells were subcutaneously injected into the right flank of each mouse. When the tumor volume reached approximately 80 mm3, the mice were randomly grouped (*n* = 6 per group). A mixture of 10 μg TMPRSS4-specific (#665) or scrambled shRNA vector and in vivo-jetPEI transfection reagent was intratumorally injected into mice at an interval of 2 or 3 days (total of 10 times). Tumor volume and body weight were measured for 39 days. Value of the minimum per group was excluded for the mean calculation. Values represent mean ± standard deviation (SD). *P < 0.05; **P <. 0.01; ***P < 0.001. **Supplementary Figure S3.** Suppression of TMPRSS4 reduces ALDH activity in HT-29 and HCT-116 cells. Cells were transfected with TMPRSS4-specific shRNA vectors for 48 h. Transfected cells were subjected to ALDH assay (A and C) or lysed for immunoblot analysis (B and D). Densitometric quantification was performed on the immunoblots using GAPDH as a loading control. The mean relative density from three independent experiments is shown under the immunoblots. Values represent mean ± SD. ***P < 0.001. **Supplementary Figure S4.** AP-1 and SP1 are involved in TMPRSS4-induced upregulation of SLUG and TWIST1. (A, B) PC3 cells were co-transfected with TMPRSS4 expression vector and siRNA specific to c-Jun (a main component of AP-1) or SP1 for 48 h. Transfected cells were lysed for real-time qPCR analysis (A) and immunoblot analysis (B). Anti-myc antibody was used to detect myc-tagged TMPRSS4 (B). Values represent mean ± SD. ***P < 0.001 compared with vector + control siRNA; §§§*P* < 0.001 compared with TMPRSS4 + control siRNA. (C) PC3 cells were transfected with c-Jun or SP1 expression vector for 48 h and lysed for immunoblot analysis. Densitometric quantification was performed on the immunoblots using GAPDH as a loading control. The mean relative density from three independent experiments is shown under the immunoblots. **Supplementary Figure S5.** SOX2 is required for TMPRSS4-induced cancer stem–like features. PC3 cells were co-transfected with a TMPRSS4 expression vector and siRNA specific to SOX2 for 48 h. Transfected cells were subjected to tumorsphere formation (A), cell survival (B), and anoikis (C) assays. Values represent mean SD. *P < 0.05; **P < 0.01; ***P < 0.001 compared with vector + control siRNA; §§*P* < 0.01; §§§*P* < 0.001 compared with TMPRSS4 + control siRNA. **Supplementary Figure S6.** SLUG and TWIST1 did not substantially change TMPRSS4 expression. The Endogenous TMPRSS4 level was analyzed in whole-cell lysates from Fig. 5 A and B and Fig. 6A. Densitometric quantification was performed on the immunoblots using GAPDH as a loading control. The mean relative density from three independent experiments is shown under the immunoblots. **Supplementary Figure S7.** Suppression of SOX2 did not substantially affect the expression of SLUG, TWIST1, or stemness-related factors. Cells were transfected with siRNAs specific to SOX2 (#2; 5′-CAGUACAACUCCAUGACCA-3′ and #3; 5′-GCUCUUGGCUCCAUGGGUU-3′) for 48 h and then lysed for immunoblot analysis. GAPDH was used as an internal control. Densitometric quantification was performed on the immunoblot using GAPDH as a loading control. The mean relative density from three independent experiments is shown under the immunoblots. SOX2 suppression did not substantially affect expression of SLUG, TWIST1, CD133, or BMI1 (moderately reduced in HEK293E cells), in PC3 and HEK293E cells.

## Data Availability

The datasets used and/or analyzed during the current study are available from the corresponding author on reasonable request.

## Abbreviations

ALDH: aldehyde dehydrogenase
CCLE: Cancer Cell Line Encyclopedia
CSC: Cancer stem cell
CTC: Circulating tumor cell
EMT: Epithelial–mesenchymal transition
HEK293E: Human embryonic kidney 293E
PTGER2: Prostaglandin E receptor 2
qPCR: Quantitative polymerase chain reaction
shRNA: Short hairpin RNA
siRNA: Small interfering RNA
TCGA: The Cancer Genome Atlas
TTSP: Type II transmembrane protease
