# Supplementary material for: TMPRSS4 promotes cancer stem–like properties in prostate cancer cells through upregulation of SOX2 by SLUG and TWIST1
Source: J Exp Clin Cancer Res. 2021 Nov 22;40:372. doi: 10.1186/s13046-021-02147-7 (PMC8607621; doi:10.1186/s13046-021-02147-7)

TMPRSS4 promotes cancer stem–like properties in prostate cancer cells through upregulation of SOX2 by SLUG and TWIST1

Yunhee Lee, Junghwa Yoon, Dongjoon Ko, Minyeong Yu, Soojin Lee, Semi Kim

Supplementary Figure Legends

Supplementary Figure S1. **TMPRSS4 promotes ALDH activation in DU145 cells.** Transfected DU145 cells were incubated with ALDH substrate for 30 min and then analyzed by flow cytometry. Cells were quenched with DEAB, an ALDH inhibitor, as a negative control.

Supplementary Figure S2. **Effects of TMPRSS4 suppression in 22Rv1 and LNCaP clone FGC cells *in vitro* and *in vivo*.** (A-D, F-H) Cells were transfected with TMPRSS4-specific shRNA vector (#665) for 48 h. Transfected cells were subjected to tumorsphere formation (A and F), cell survival (B and G), anoikis (C, H), and cell adhesion (D) assays. The number of spheroids >75 μm (for 22Rv1 cells) or >25 μm (for LNCaP-FGC cells) in diameter was counted after 10 days. (E) *In vivo* tumor growth analysis. 22Rv1 (5×10^6) cells were subcutaneously injected into the right flank of each mouse. When the tumor volume reached approximately 80 mm^3, the mice were randomly grouped (n = 6 per group). A mixture of 10 μg TMPRSS4-specific (#665) or scrambled shRNA vector and *in vivo*-jetPEI transfection reagent was intratumorally injected into mice at an interval of 2 or 3 days (total of ten times). Tumor volume and body weight were measured for 39 days. Value of the minimum per group was excluded for the mean calculation. Values represent mean ± standard deviation (SD). $^*P < 0.05$; $^{**}P <$

0.01; ***$P < 0.001$.

Supplementary Figure S3. **Suppression of TMPRSS4 reduces ALDH activity in HT-29 and HCT-116 cells.** Cells were transfected with TMPRSS4-specific shRNA vectors for 48 h. Transfected cells were subjected to ALDH assay (A and C) or lysed for immunoblot analysis (B and D). Densitometric quantification was performed on the immunoblots using GAPDH as a loading control. The mean relative density from three independent experiments is shown under the immunoblots. Values represent mean ± SD. ***$P < 0.001$.

Supplementary Figure S4. **AP-1 and SP1 are involved in TMPRSS4-induced upregulation of SLUG and TWIST1.** (A, B) PC3 cells were co-transfected with TMPRSS4 expression vector and siRNA specific to c-Jun (a main component of AP-1) or SP1 for 48 h. Transfected cells were lysed for real-time qPCR analysis (A) and immunoblot analysis (B). Anti-myc antibody was used to detect myc-tagged TMPRSS4 (B). Values represent mean ± SD. ***$P <$ 0.001 compared with vector + control siRNA; $^{§§§}P < 0.001$ compared with TMPRSS4 + control siRNA. (C) PC3 cells were transfected with c-Jun or SP1 expression vector for 48 h and lysed for immunoblot analysis. Densitometric quantification was performed on the immunoblots using GAPDH as a loading control. The mean relative density from three independent experiments is shown under the immunoblots.

Supplementary Figure S5. **SOX2 is required for TMPRSS4-induced cancer stem–like features.** PC3 cells were co-transfected with a TMPRSS4 expression vector and siRNA specific to SOX2 for 48 h. Transfected cells were subjected to tumorsphere formation (A), cell

survival (B), and anoikis (C) assays. Values represent mean ± SD. *$P < 0.05$; **$P < 0.01$; ***$P < 0.001$ compared with vector + control siRNA; $^{§§}P < 0.01$; $^{§§§}P < 0.001$ compared with TMPRSS4 + control siRNA.

Supplementary Figure S6. SLUG and TWIST1 did not substantially change TMPRSS4 expression. The Endogenous TMPRSS4 level was analyzed in whole-cell lysates from Figure 5A and B and Figure 6A. Densitometric quantification was performed on the immunoblots using GAPDH as a loading control. The mean relative density from three independent experiments is shown under the immunoblots.

Supplementary Figure S7. **Suppression of SOX2 did not substantially affect the expression of SLUG, TWIST1, or stemness-related factors.** Cells were transfected with siRNAs specific to SOX2 (#2; 5′-CAGUACAACUCCAUGACCA-3′ and #3; 5′-GCUCUUGGCUCCAUGGGUU-3′) for 48 h and then lysed for immunoblot analysis. GAPDH was used as an internal control. Densitometric quantification was performed on the immunoblot using GAPDH as a loading control. The mean relative density from three independent experiments is shown under the immunoblots. SOX2 suppression did not substantially affect expression of SLUG, TWIST1, CD133, or BMI1 (moderately reduced in HEK293E cells), in PC3 and HEK293E cells.

Supplementary Figure S1. Lee et al.

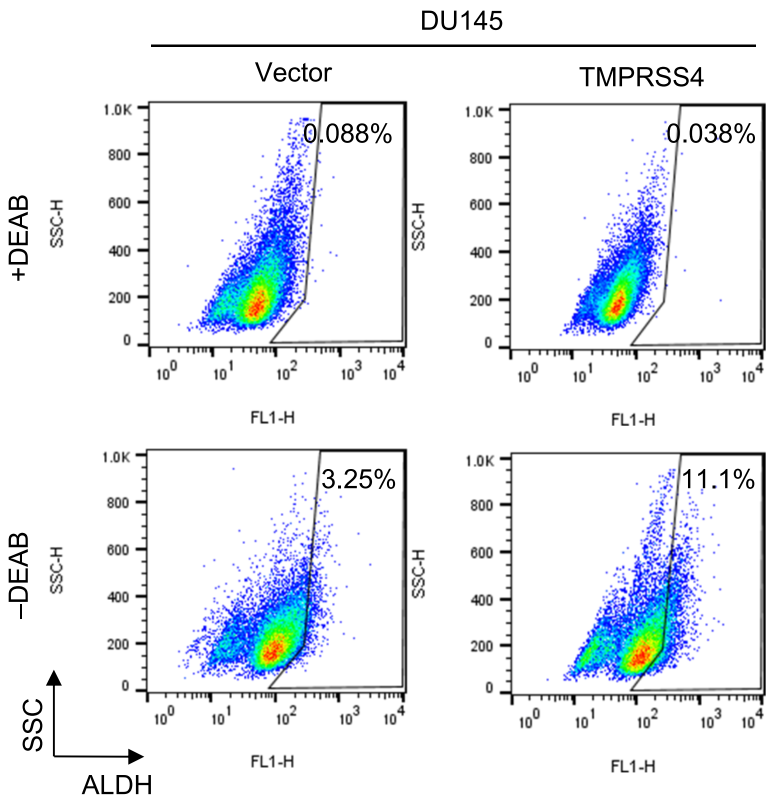

Supplementary Figure S2. Lee et al.

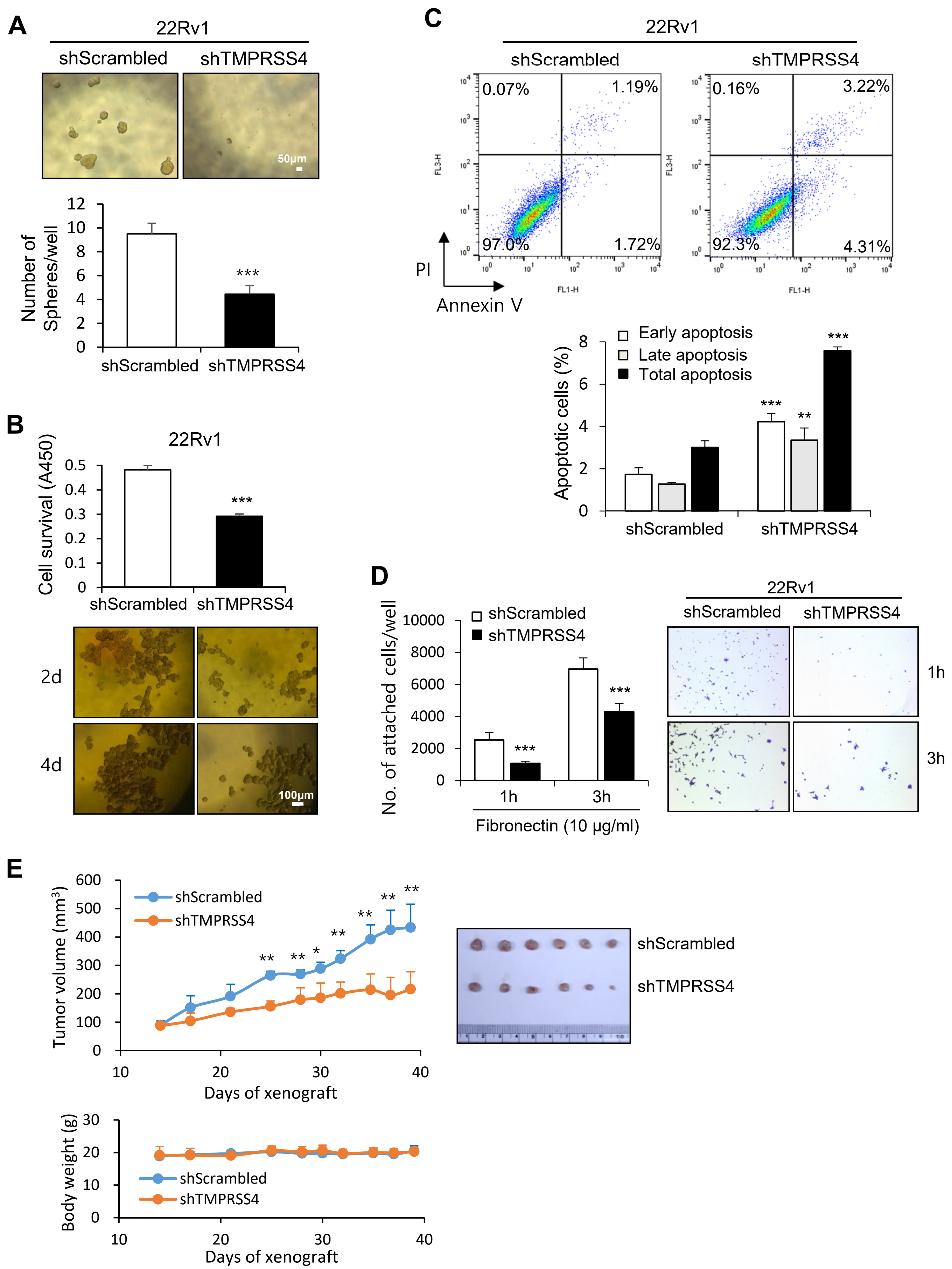

Supplementary Figure S2. Lee et al. (continued)

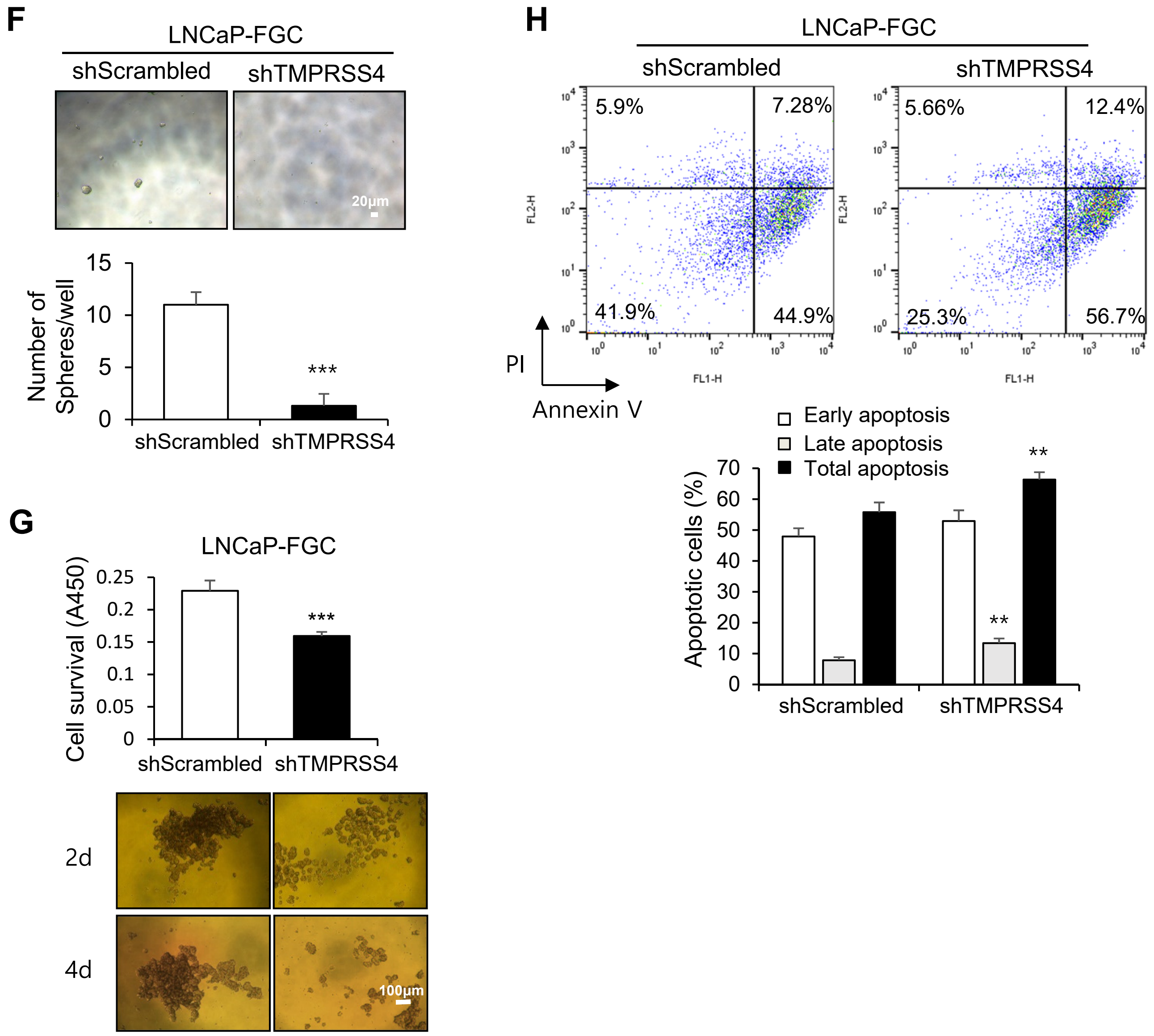

Supplementary Figure S3. Lee et al.

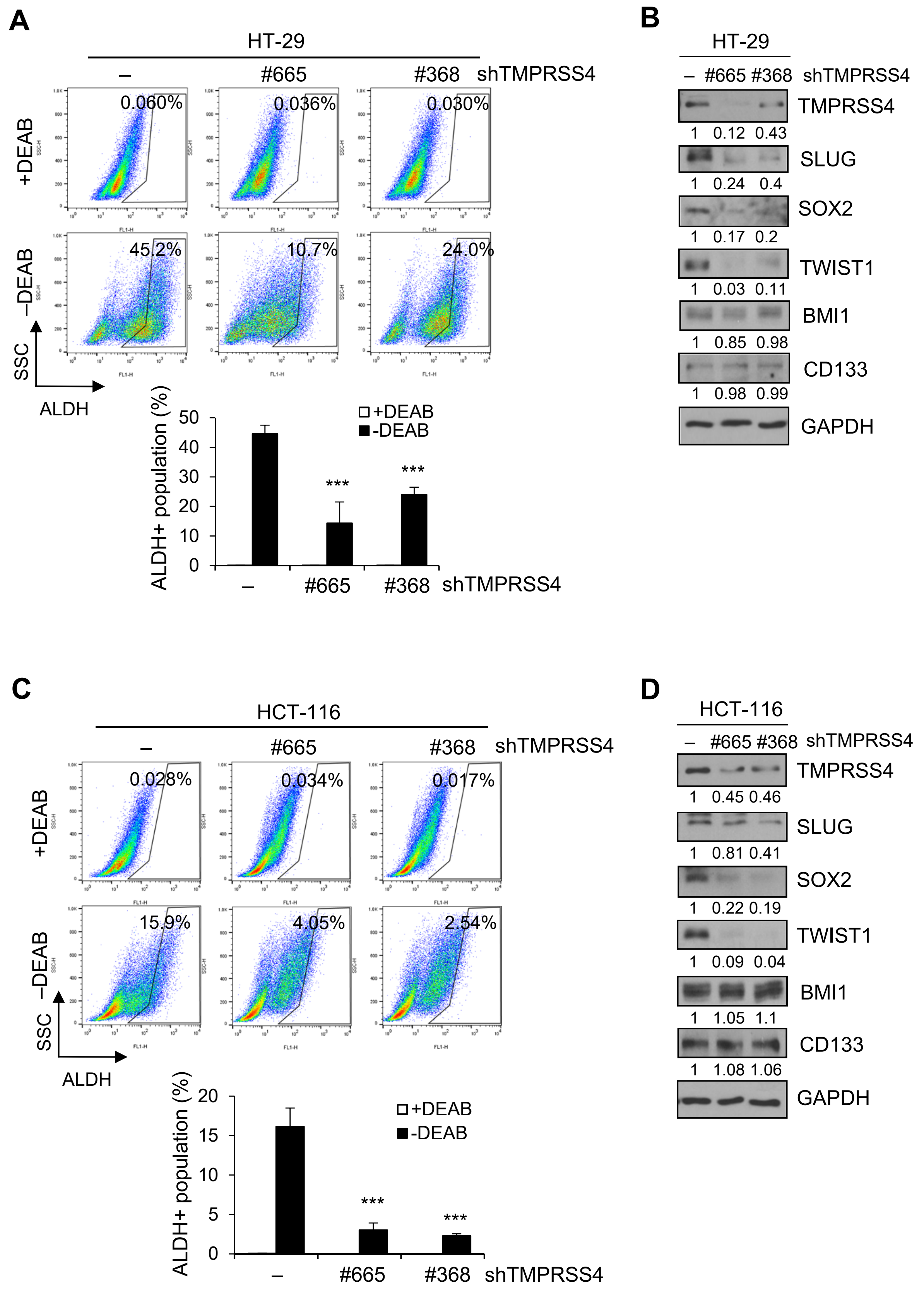

Supplementary Figure S4. Lee et al.

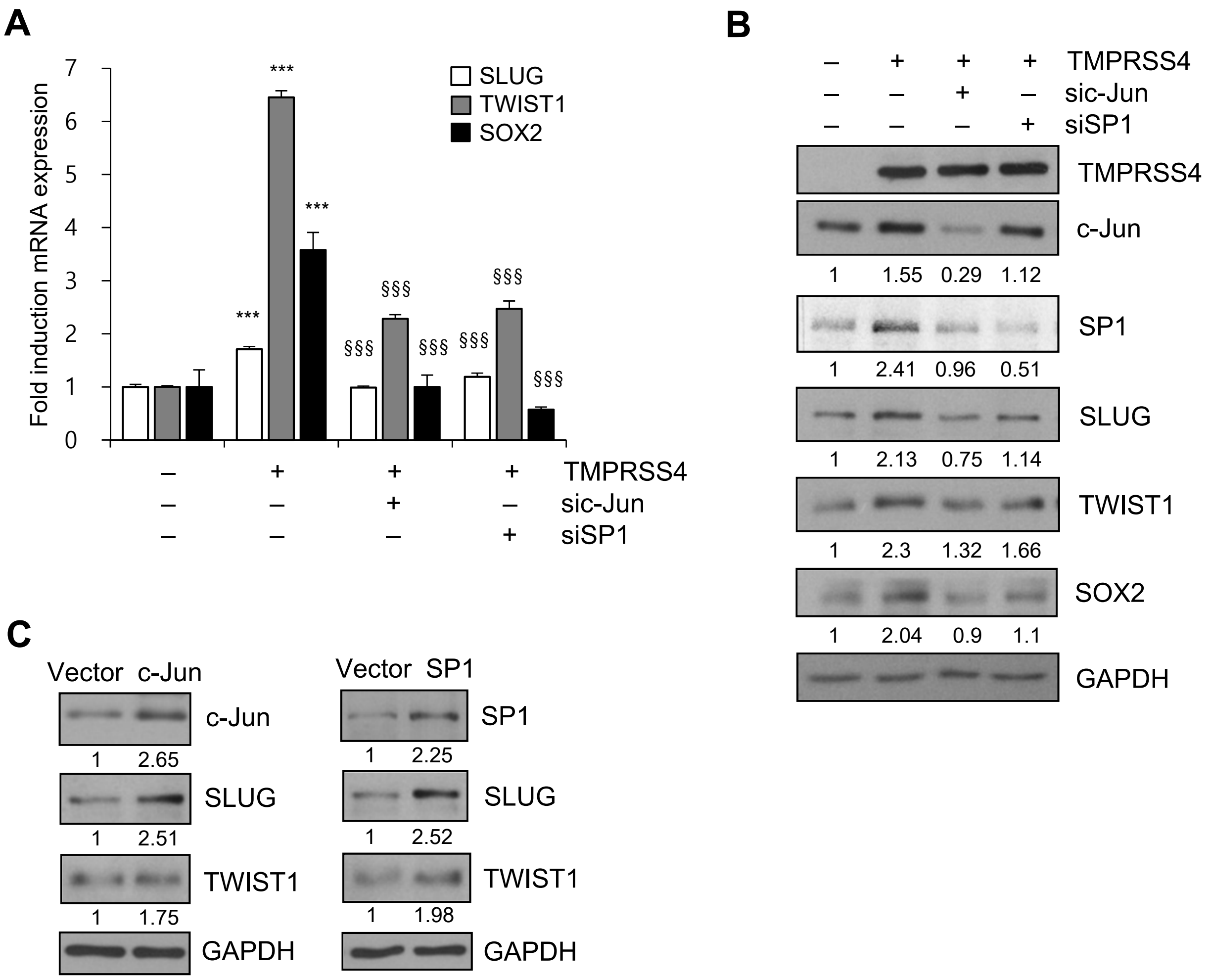

Supplementary Figure S5. Lee et al.

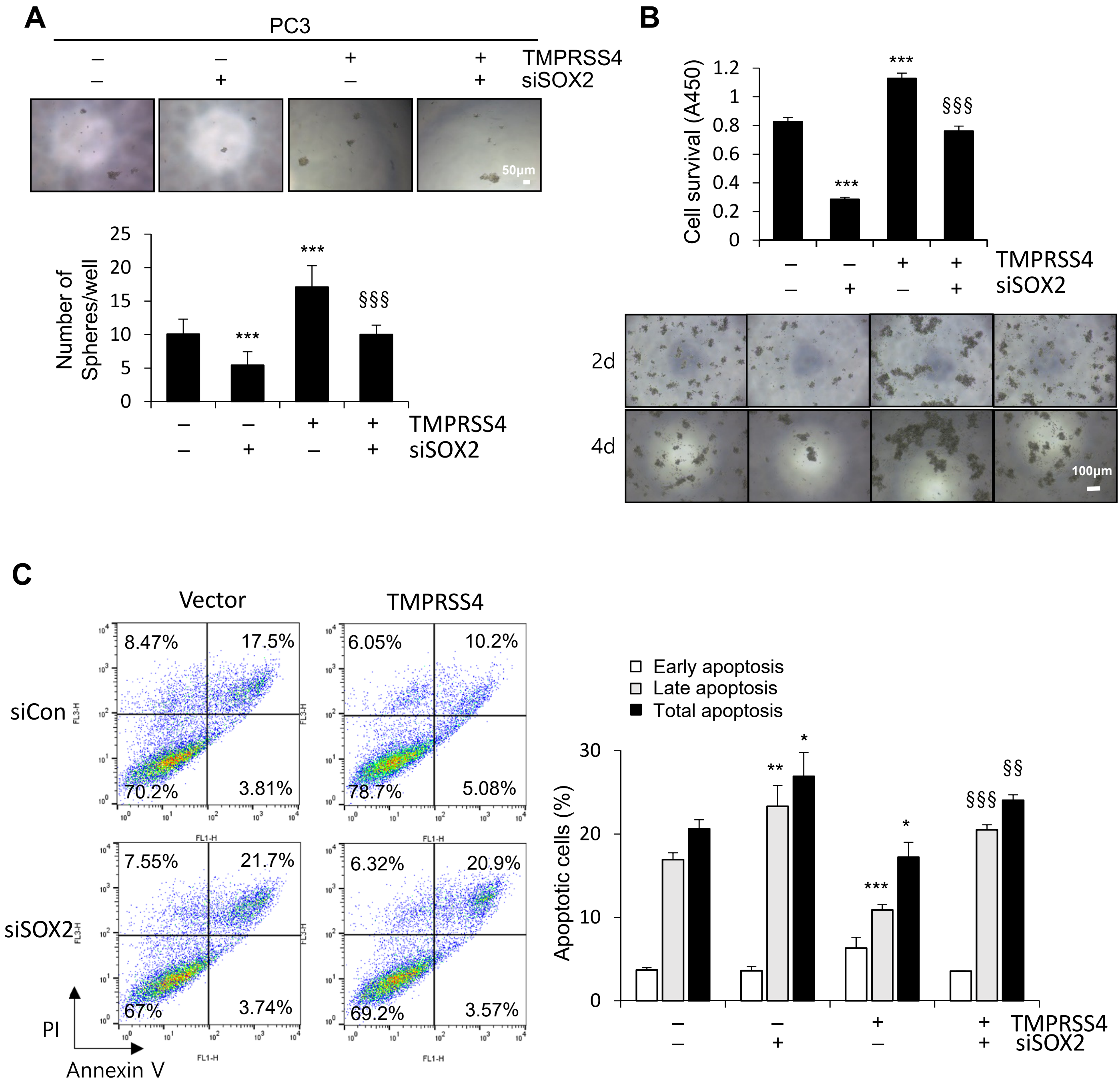

Supplementary Figure S6. Lee et al.

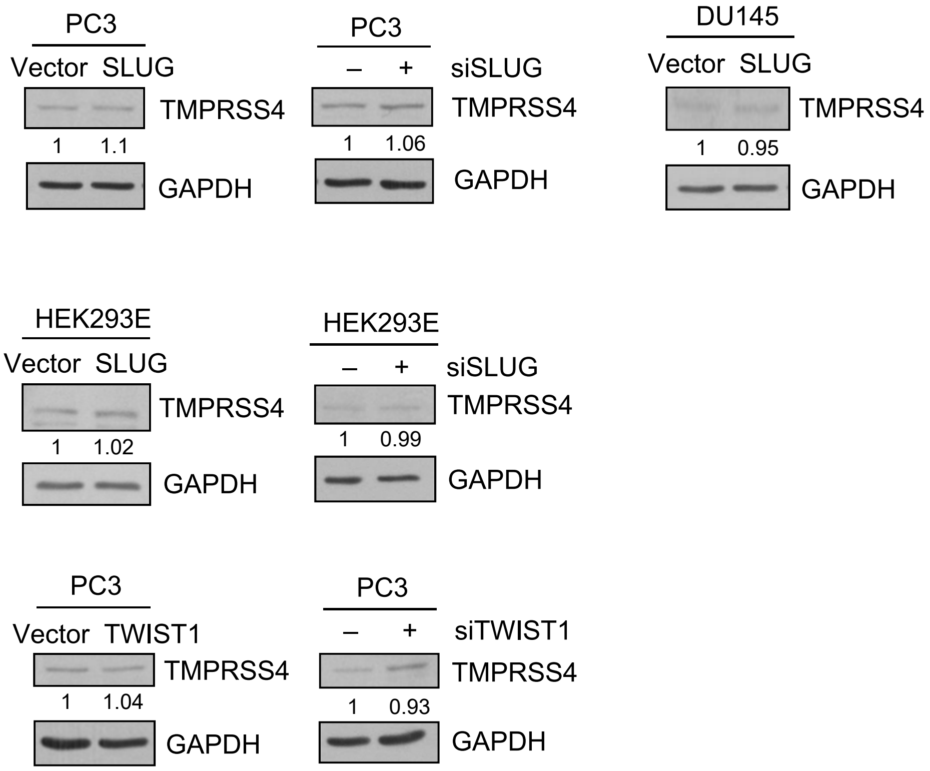

Supplementary Figure S7. Lee et al.

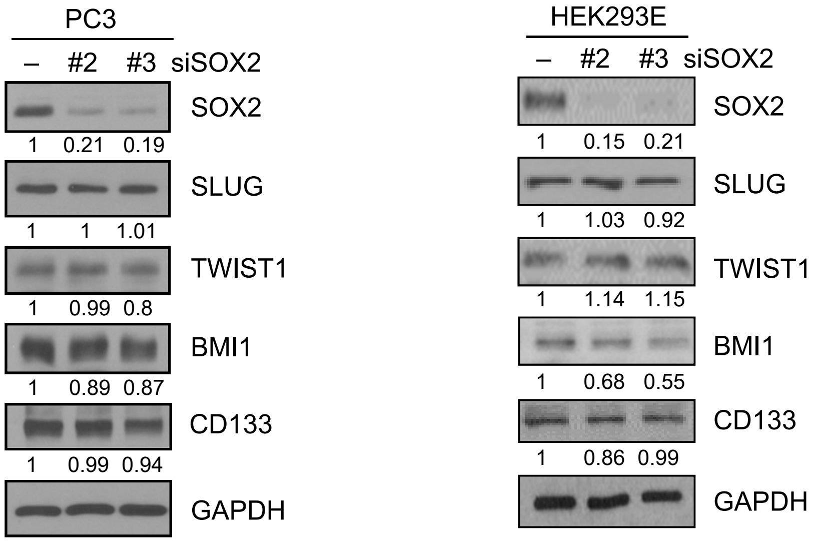

Supplement: Supplementary file 1 — Additional file 1: Supplementary Figure S1. TMPRSS4 promotes ALDH activation in DU145 cells. Transfected DU145 cells were incubated with ALDH substrate for 30 min and then analyzed by flow cytometry. Cells were quenched with DEAB, an ALDH inhibitor, as a negative control. Supplementary Figure S2. Effects of TMPRSS4 suppression in 22Rv1 and LNCaP clone FGC cells in vitro and in vivo. (A-D, F-H) Cells were transfected with TMPRSS4-specific shRNA vector (#665) for 48 h. Transfected cells were subjected to tumorsphere formation (A and F), cell survival (B and G), anoikis (C, H), and cell adhesion (D) assays. The number of spheroids > 75 μm (for 22Rv1 cells) or > 25 μm (for LNCaP-FGC cells) in diameter was counted after 10 days. (E) In vivo tumor growth analysis. 22Rv1 (5 × 10^6^) cells were subcutaneously injected into the right flank of each mouse. When the tumor volume reached approximately 80 mm3, the mice were randomly grouped (n = 6 per group). A mixture of 10 μg TMPRSS4-specific (#665) or scrambled shRNA vector and in vivo-jetPEI transfection reagent was intratumorally injected into mice at an interval of 2 or 3 days (total of 10 times). Tumor volume and body weight were measured for 39 days. Value of the minimum per group was excluded for the mean calculation. Values represent mean ± standard deviation (SD). *P < 0.05; **P <. 0.01; ***P < 0.001. Supplementary Figure S3. Suppression of TMPRSS4 reduces ALDH activity in HT-29 and HCT-116 cells. Cells were transfected with TMPRSS4-specific shRNA vectors for 48 h. Transfected cells were subjected to ALDH assay (A and C) or lysed for immunoblot analysis (B and D). Densitometric quantification was performed on the immunoblots using GAPDH as a loading control. The mean relative density from three independent experiments is shown under the immunoblots. Values represent mean ± SD. ***P < 0.001. Supplementary Figure S4. AP-1 and SP1 are involved in TMPRSS4-induced upregulation of SLUG and TWIST1. (A, B) PC3 cells were co-tran [file 13046_2021_2147_MOESM1_ESM.pdf]
